# Supplementary material for: Acute Iron Deprivation Reprograms Human Macrophage Metabolism and Reduces Inflammation In Vivo
Source: Cell Rep. 2019 Jul 9;28(2):498–511.e5. doi: 10.1016/j.celrep.2019.06.039 (PMC6635384; doi:10.1016/j.celrep.2019.06.039)
Supplement: Document S1. Figures S1–S6 and Tables S1 and S2 [file mmc1.pdf]

Cell Reports, Volume *28*

Supplemental Information

Acute Iron Deprivation Reprograms Human Macrophage Metabolism and Reduces Inflammation *In Vivo*

Marie Pereira, Tai-Di Chen, Norzawani Buang, Antoni Olona, Jeong-Hun Ko, Maria Prendecki, Ana S.H. Costa, Efterpi Nikitopoulou, Laura Tronci, Charles D. Pusey, H. Terence Cook, Stephen P. McAdoo, Christian Frezza, and Jacques Behmoaras

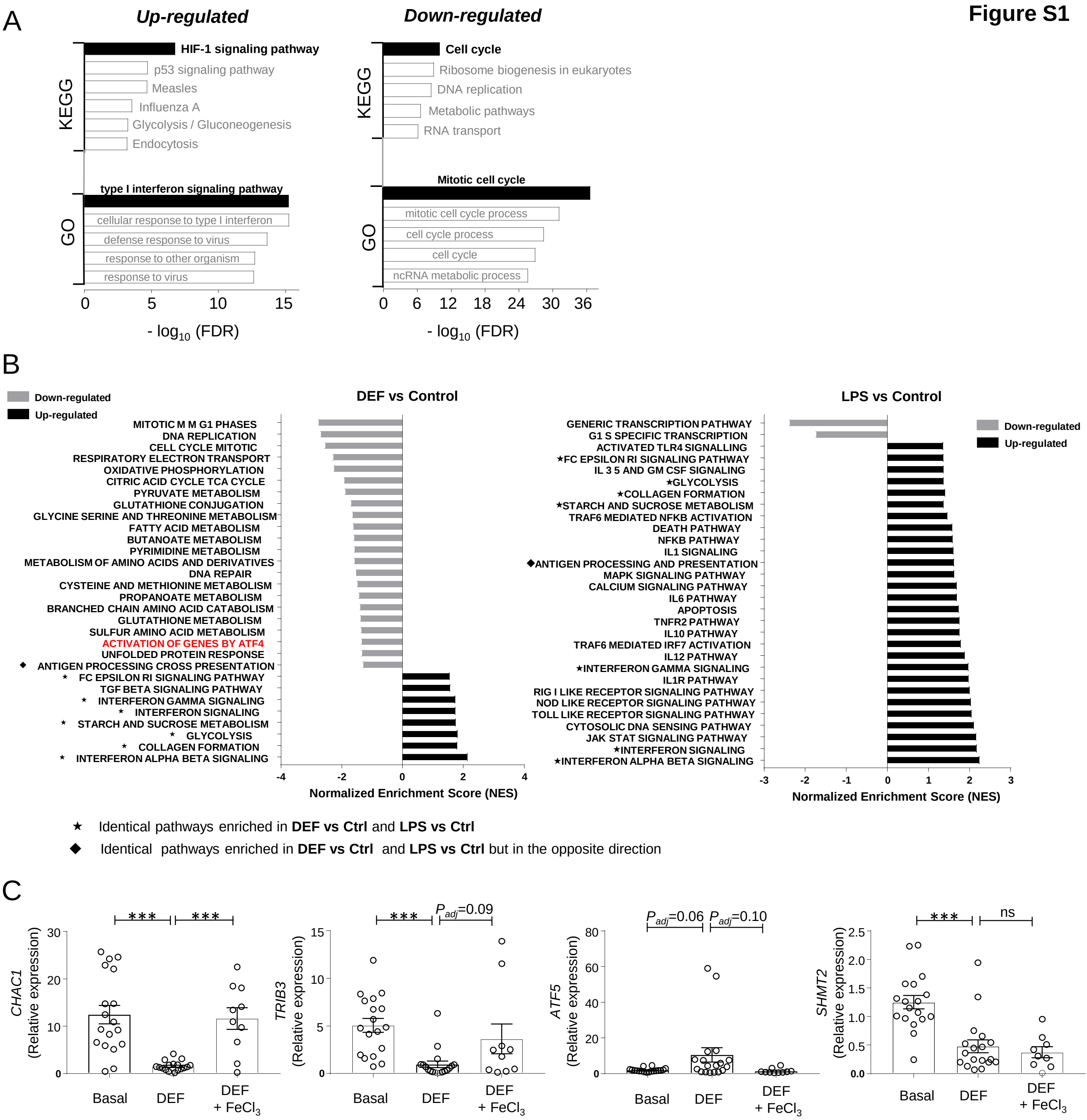

Figure S1. **Acute iron deprivation causes a distinct transcriptome and mediates an ATF4-dependent transcriptional response in human macrophages**. Related to Figure 1. (A) KEGG and Gene ontology (GO) analyses on differentially expressed genes (DEF vs Ctrl) in human macrophages. (B) Gene Set Enrichment Analysis (GSEA), on DEF (500μM, 24h) vs Ctrl and LPS (3h, 100ng/ml) vs Ctrl comparisons in human macrophages. 'Activation of genes by ATF4' pathway is shown in red. (C) ATF4 target gene expression in iron-deprived (DEF) and rescued (DEF+$FeCl_3$) human macrophages. ***, P<0.001 by ANOVA followed by Tukey's multiple comparisons test. P_{adj} denotes adjusted *P*-value. FDR, false discovery rate. Error bars represent SEM.

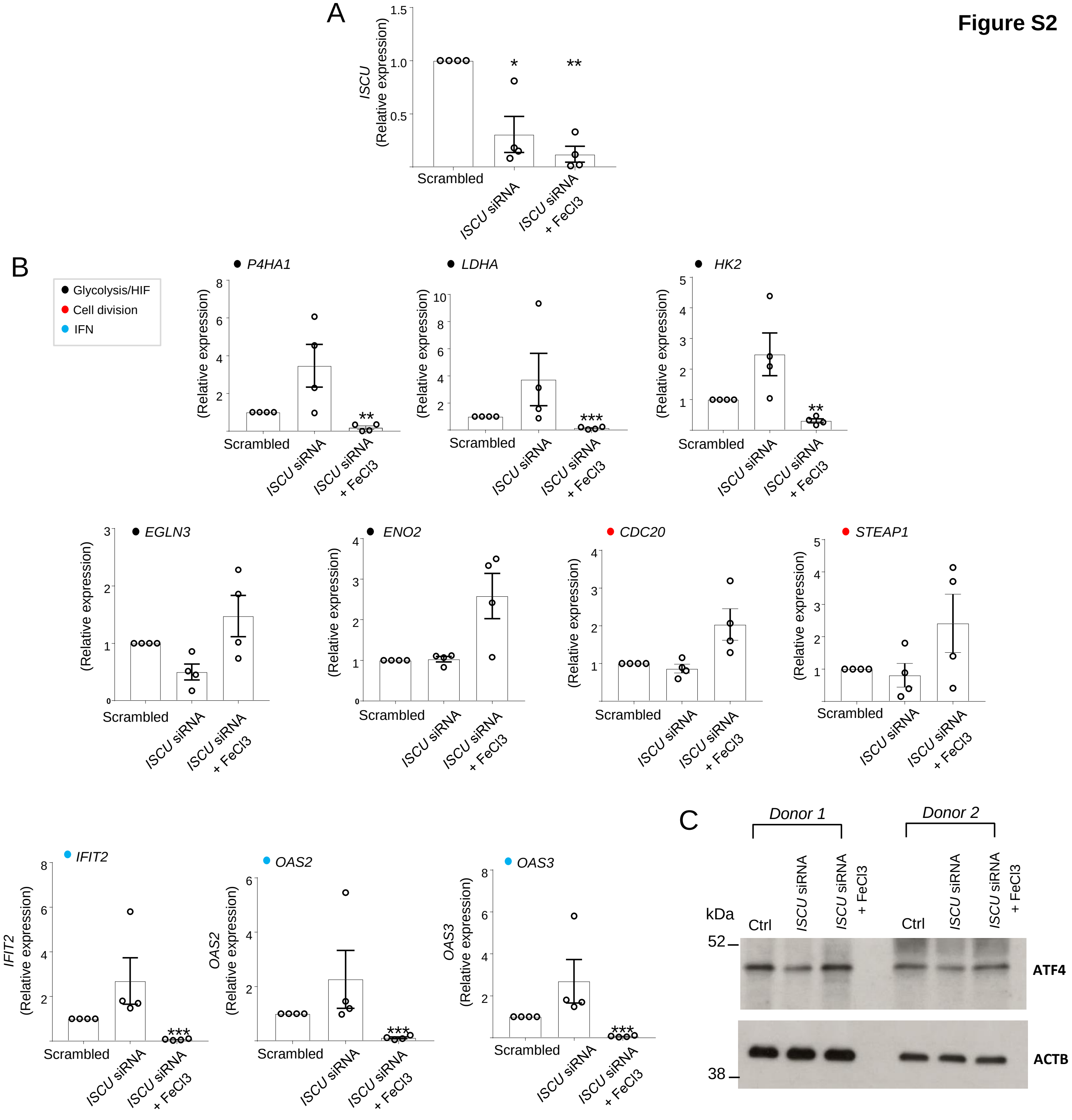

Figure S2. ***ISCU* mRNA knockdown causes similar transcriptional responses to the ones following deferiprone stimulation in human macrophages**. Related to Figure 1C and 1D. (A) *ISCU* expression by qRT-PCR. Human macrophages from 4 donors (n=4) were either treated with *ISCU* or scrambled siRNA. Iron replenishment was obtained by adding 200 µM $FeCl_3$ for 8 hours (*ISCU* siRNA + $FeCl_3$) following *ISCU*-siRNA. (B) qRT-PCR for glycolysis/HIF, cell cycle/mitosis and IFN genes (colour-coded). (C) ATF4 Western Blotting in *ISCU* siRNA and ISCU siRNA followed by iron replenishment (*ISCU* siRNA + $FeCl_3$) in 2 donors. *, P<0.05; **, P<0.01, ***, P<0.001 by one sample t-test compared to 100% (Scrambled). Error bars represent SEM.

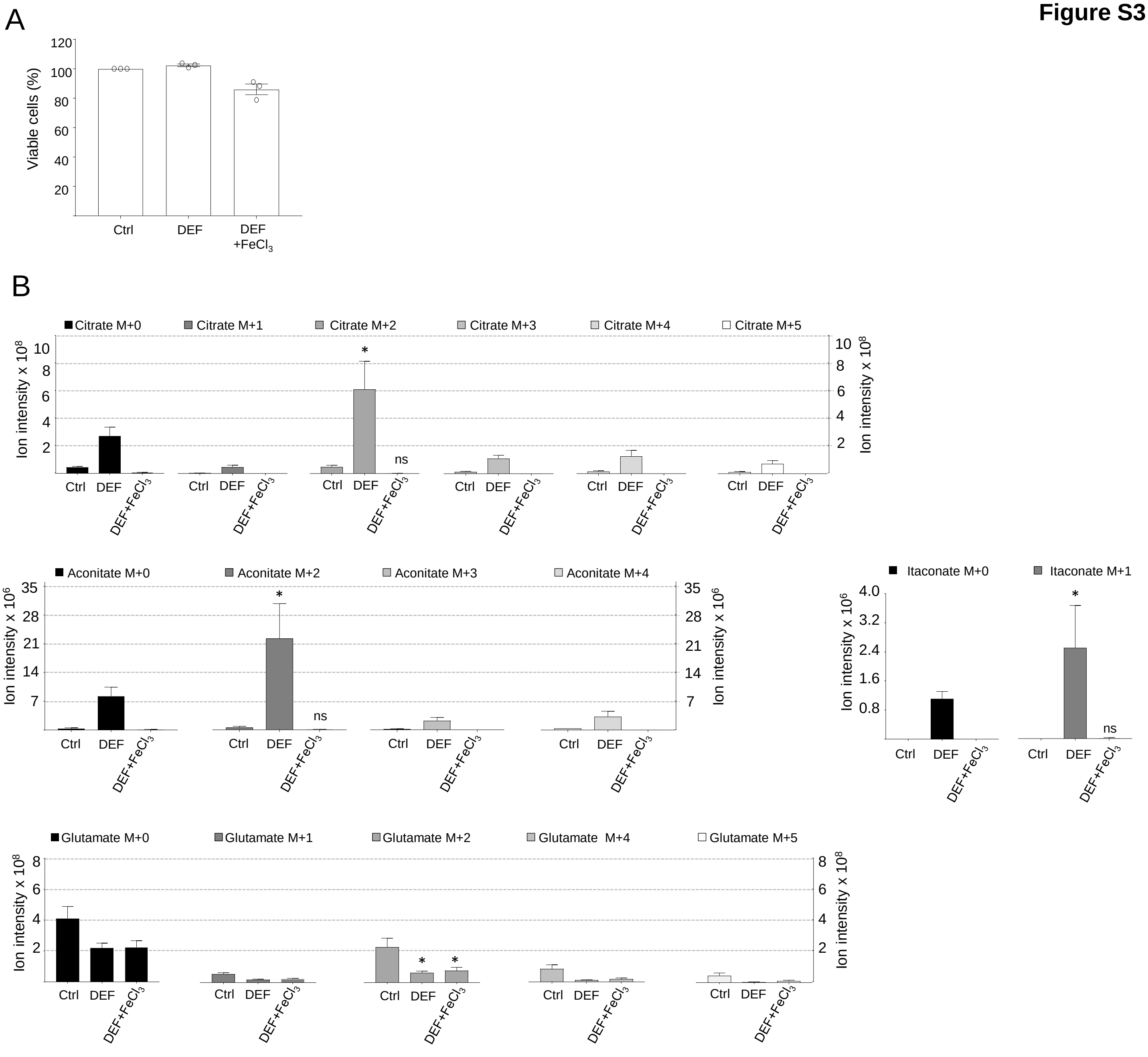

Figure S3. **Acute iron deprivation causes citrate, aconitate and itaconate accumulation while decreasing glutamate levels in human macrophages**. Related to Figure 3. (A) Cell viability measured by Alamar blue. n=3 donors. (B) Isotopologue quantification by LC-MS for citrate, aconitate, itaconate and glutamate. n=6 donors. *, $P<0.05$, when compared to control by ANOVA followed by Dunnett's multiple comparisons test. For simplicity, only glucose-derived isotopologues were compared for significance (M+2 citrate, glutamate, aconitate and M+1 itaconate). Error bars represent SEM.

Figure S4

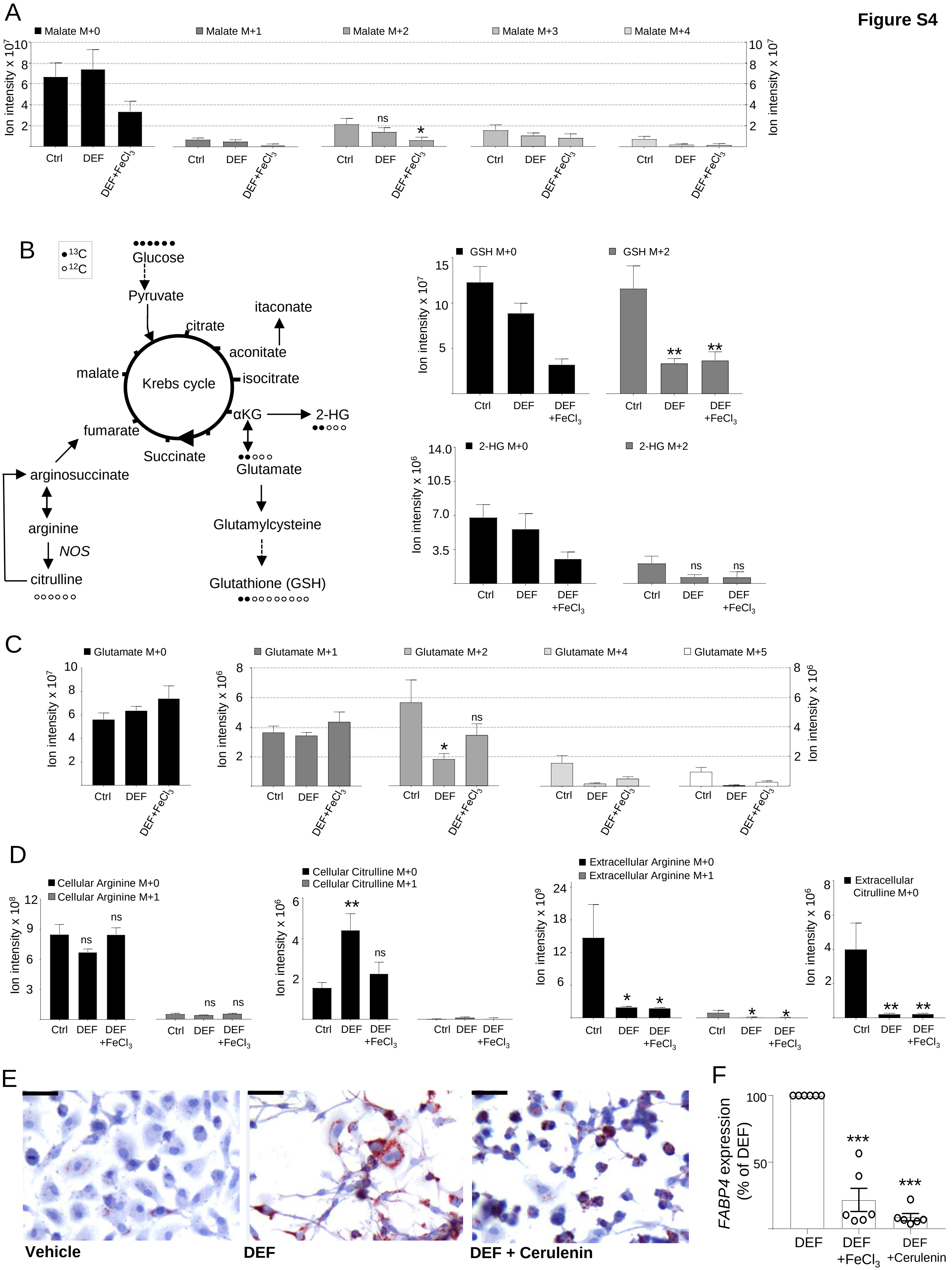

A
Malate M+0
Malate M+1
Malate M+2
Malate M+3
Malate M+4
Ion intensity x 10^7
Ctrl
DEF
DEF+FeCl3
ns
*
B
13C
12C
Glucose
Pyruvate
citrate
itaconate
aconitate
isocitrate
malate
Krebs cycle
αKG
2-HG
fumarate
Succinate
Glutamate
arginosuccinate
arginine
NOS
Glutamylcysteine
citrulline
Glutathione (GSH)
GSH M+0
GSH M+2
2-HG M+0
2-HG M+2
C
Glutamate M+0
Glutamate M+1
Glutamate M+2
Glutamate M+4
Glutamate M+5
Ion intensity x 10^6
D
Cellular Arginine M+0
Cellular Arginine M+1
Ion intensity x 10^8
Cellular Citrulline M+0
Cellular Citrulline M+1
Extracellular Arginine M+0
Extracellular Arginine M+1
Ion intensity x 10^9
Extracellular Citrulline M+0
E
Vehicle
DEF
DEF + Cerulenin
F
FABP4 expression (% of DEF)
DEF
DEF +FeCl3
DEF +Cerulenin
***

Figure S4. **Acute iron deprivation causes a brake in the TCA cycle, alters the urea cycle and induces *de novo* lipid accumulation**. Related to Figure 3. (A) Quantification of malate isotopologues by LC-MS in control (Ctrl), iron-deprived (DEF, 500µM), iron-rescued human macrophages with 200µM $FeCl_3$ supplementation (DEF+$FeCl_3$, 8h); n=6 donors. (B) Schematic illustration of glucose-αKG-derived metabolites and urea cycle in uniformly labelled [U]-^{13}C- glucose catabolism (left panel) and glutathione (GSH), 2-hydroxyglutarate (2-HG) M+0 and M+2 isotopologues (right panel) in control (Ctrl), iron-deprived (DEF), iron-rescued human macrophages with $FeCl_3$ supplementation (DEF+$FeCl_3$, 8h); n=6 donors. (C) Extracellular glutamate isotopologues quantified by LC-MS; n=6 donors. (D) Cellular (upper panel) and extracellular (lower panel) citrulline and arginine isotopologues quantified by LC-MS; n=6 donors. (E) Vehicle, DEF (500µM) or concomitant DEF and cerulenin (20µM)-treated human macrophages stained for oil-red-o. (F) *FABP4* expression (% of DEF) in hMDMs (n=2 donors). In (A), (B) and (C), only glucose-derived M+2 istopologues were compared for significance (M+2 GSH, M+2 2-HG, M+2 glutamate). *, $P<0.05$; **, $P<0.01$ compared to Control (Ctrl) by ANOVA followed by Dunnett's multiple comparisons test. ***, $P<0.001$ by one sample t-test compared to 100% (DEF). ns, non-significant. Scale bars, 40µm. Error bars represent SEM.

Figure S5

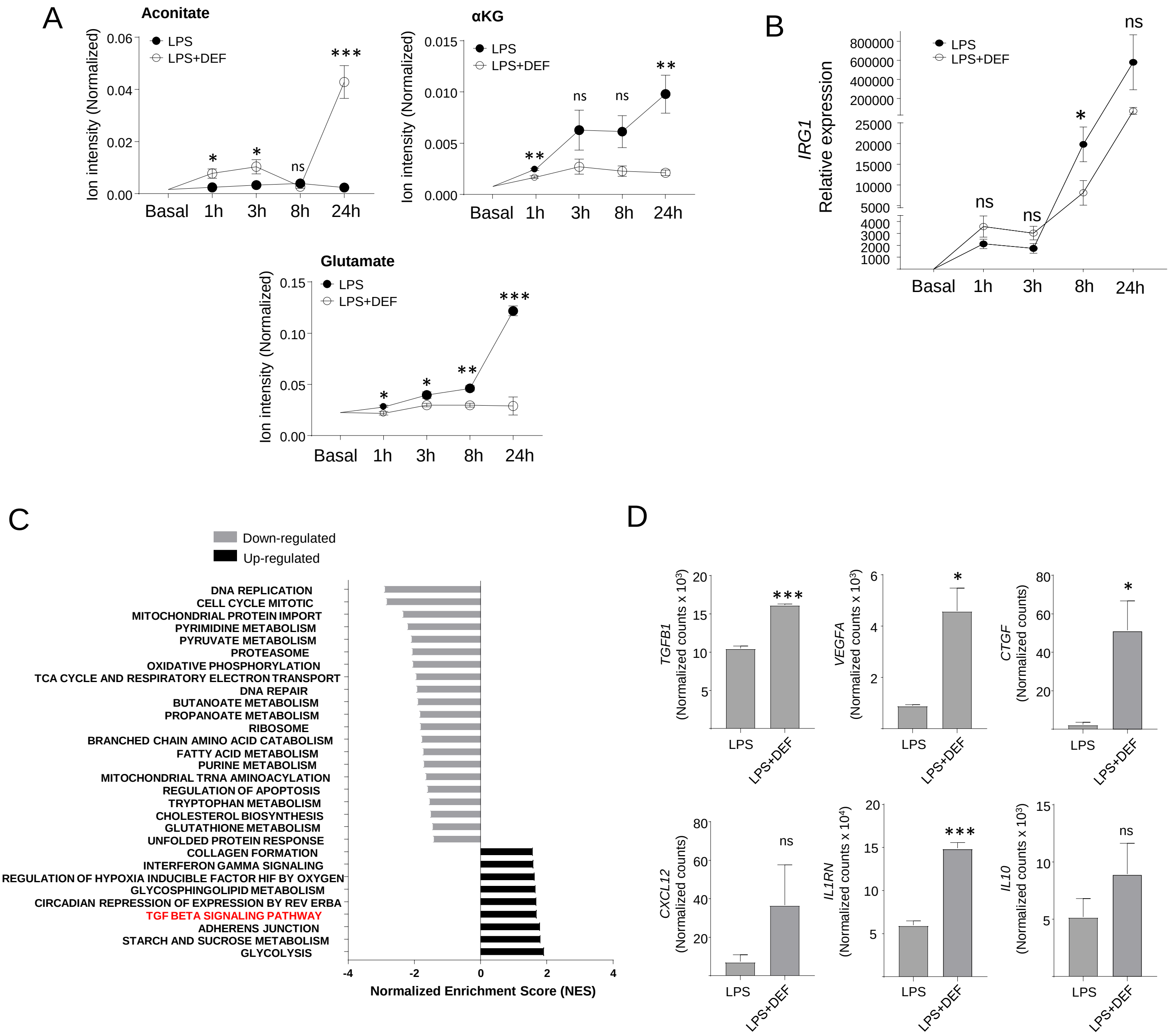

Figure S5. **Acute iron chelation limits pro-inflammatory macrophage polarization and promotes TGF-β signalling pathway**. Related to Figure 4. (A) LC-MS for aconitate, α-ketoglutarate (αKG) and glutamate in basal and LPS-treated (100ng/ml) human macrophages throughout a time course. LPS+DEF refers to macrophages pre-treated with DEF (500µM, 24h) prior to the LPS time-course; n=4 donors/group. (B) *IRG1* mRNA measured by qRT-PCR in basal and DEF-treated cells (500µM, 24h) throughout an LPS (100ng/ml) time course; n=4 donors/group. (C) Gene Set Enrichment Analysis for LPS+DEF vs. LPS comparison. (LPS, 3h, 100ng/ml; DEF, 24h, 500µM). TGFβ signalling pathway is shown in red. (D) *TGFB1*, *VEGFA*, *CTGF*, *CXCL12*, *IL1RN*, *IL10* expression measured by RNA-seq (normalized counts) in LPS and LPS+DEF stimulated human macrophages; n=3 donors/group. *, $P<0.05$; **, $P<0.01$; ***, $P<0.001$ by t-test; ns, non-significant. Error bars represent SEM.

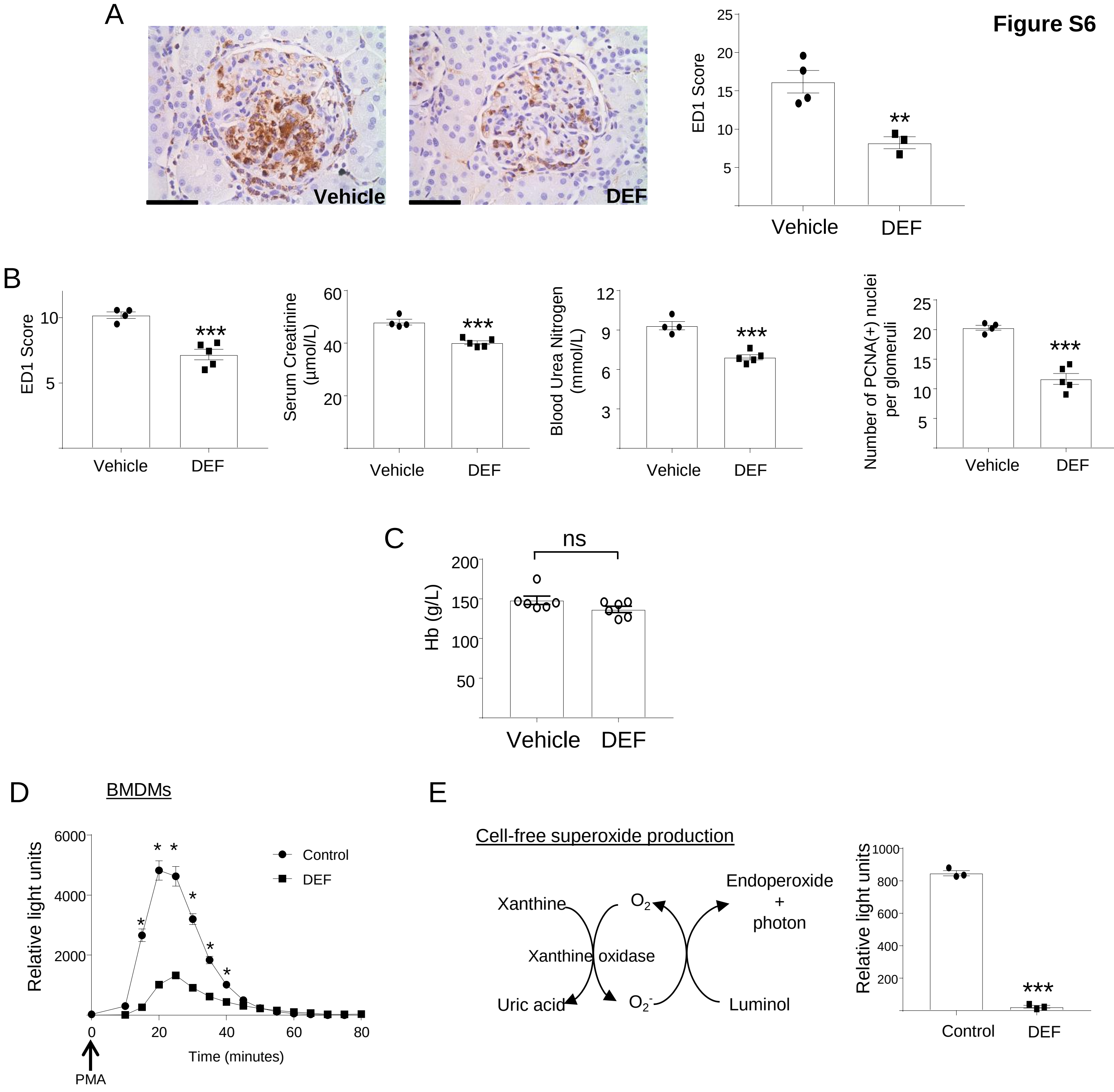

Figure S6. **Acute iron chelation decreases the severity of glomerulonephritis and reduces superoxide production in macrophages**. Related to Figure 6. (A) ED1 staining showing macrophages in the glomeruli; ED1 score (right panel) was normalized to glomerular size; at least n=3 rats/group; preventive NTN experiment. (B) ED1 score, serum creatinine, blood urea nitrogen, glomerular PCNA+ nuclei; at least n=4 rats/group; therapeutic NTN experiment. (C) Serum haemoglobin (Hb) counts; n=6 rats/group. (D) Superoxide chemiluminescence signal in WKY BMDMs stimulated with 1 µM phorbol myristate acetate (PMA) with or without of deferiprone (DEF, 500 µM); at least 3 rats/group. (E) Superoxide chemiluminescence signal produced by xanthine-xanthine oxidase reaction in a cell free setup (left panel) with or without deferiprone (DEF, 500 µM, right panel). *, P<0.05; **, P<0.01; ***, P<0.001 by t-test. ns, non-significant. Scale bars, 40µ. Error bars represent SEM.

	Patient 1	*Patient 2*	*Patient 3*	Normal Ranges
**Age**	77	83	51	
**Gender**	F	M	M	
**Cause of CKD**	Diabetes	Diabetes	Diabetes	
**Creatinine (μmol/L)**	272	302	456	60-125
**eGFR (ml/min/1.73m^2)**	16.2	18.5	13.9	>90
**CKD Stage**	IV	IV	V	
**Hb (g/dL)**	10.2	9.6	10.3	13-16.5
**Ferritin (μg/L)**	281	30	106	20-300
**Iron (μmol/L)**	9	N/A	11	12-31
**Transferrin Saturation (%)**	20	N/A	24	16-55
**Transferrin (g/L)**	1.8	N/A	1.8	1.7-3.4
**MCV (fL)**	98.7	87.2	89	83.5-99.5
**CRP (mg/L)**	3	19.1	0.9	0-5
**ESA**	Aranesp	No	No	

Table S1. **Clinical characteristics of chronic kidney disease (CKD) patients undergoing intravenous iron administration.** Related to Figure 1. eGFR, estimated glomerular filtration rate; Hb, haemoglobin; MCV, mean corpuscular volume; CRP, c-reactive protein; ESA, erythropoiesis stimulating agent.

Gene name / siRNA name	*Sequence*
***PLOD2*-F**	GGGAATGGACTTTTGCCGTC
***PLOD2*-R**	CCACAGCTTTCCATGACGAG
P4HA1-F	TTTGCACGGAAAGATGAGCC
***P4HA1*-R**	TCCTTCTCCACTGGCAAACA
***LDHA*-F**	GTGGAGGTTGTGCATGTTGT
***LDHA*-R**	CGTCAGAGGTGGCAGAACTA
***HK2*-F**	GCCATCCTGCAACACTTAGG
***HK2*-R**	CCACACCCACTGTCACTTTG
***STEAP1*-F**	TGCCTGGATTGAGCATGATG
***STEAP1*-R**	AATGCGTGTATTGTGCCCAG
***CDC20*-F**	ACCATGATGTTCGGGTAGCA
***CDC20*-R**	AATGTCTGCAGAGGAACCCA
***DDX21*-F**	AACTTCAAGTGGGCAAGCTG
***DDX21*-R**	CAGGGGCTACATCAGGTTCT
***EGLN3*-F**	CCTCACTGAAGACTGACCGT
***EGLN3*-R**	ACCACACACAAGACAGGGAT
***ENO2*-F**	CCCGACACCTGTATTGCATG
***ENO2*-R**	TGGGAGCCAAGAAGAGGATG
***SDHB*-F**	CACAGCTCCCCGTATCAAGA
***SDHB*-R**	CAAGAGCCACAGATGCCTTC
***NDUFS6*-F**	TCGGTTTGTAGGTCGTCAGA
***NDUFS6*-R**	CTAGTGGTGGTGCTGTCTGA
**CHAC1-F**	GACTTCATGCAGCTCTGTGG
**CHAC1-R**	TCTGTCTTGTGCACTGGAGT
***TRIB3*-F**	AGCTCACTCTGGGAACTGTG
***TRIB3*-R**	ATCCTGTCCCTCAACCTTGG
***ATF5*-F**	GGACCGCAAGCAAAAGAAGA
***ATF5*-R**	CTCGATGAGCAGGTCCTTGA
***SHMT2*-F**	CTGACTGCTCGACTTTTCCG
***SHMT2*-R**	GTGAGTAGTGGTGGTGACGA
***ISCU*-F**	ATCCTAGAAACGTGGGGTCC
***ISCU*-R**	CTTCCTCCACCGTCTTTCCT
***IFIT2*-F**	GCGAAACAACTGCTCCATCT
***IFIT2*-R**	CCAAGACATGCAAAGCCTCA
***OAS2*-F**	GCTTCCAACTCATCCACGTC
***OAS2*-R**	GGCAGGCTCAGAAGGAAAAG
***OAS3*-F**	TGCCATATTGACAGCCTCCA
***OAS3*-R**	GGATATGTGTGTGGCAGCAG
***IRG1*-F**	TGTGTGGACTTCATGAGCCT
***IRG1*-R**	TTAAACCCACCCATTCCCCA
***FABP4*-F**	AAACTGGTGGTGGAATGCGT
***FABP4*-R**	GCGAACTTCAGTCCAGGTCA
**SMARTpool siRNA *ISCU***	GAGCUAUGAGAUACGCACA<br>CAGCAUGUGGUGACGUAAU<br>CAUAAAACAGAUUGCGCAU<br>GGUCUGAAUAUUUGAUAGA

Table S2. **Primer sequences used in the study**. Related to STAR Methods. Gene names are followed by -F (Forward) and -R (Reverse). Individual siRNA target sequences are shown for the SMARTpool.
