# Supplementary material for: Acute Iron Deprivation Reprograms Human Macrophage Metabolism and Reduces Inflammation In Vivo
Source: Cell Rep. 2019 Jul 9;28(2):498–511.e5. doi: 10.1016/j.celrep.2019.06.039 (PMC6635384; doi:10.1016/j.celrep.2019.06.039)
Supplement: Document S2. Article plus Supplemental Information [file mmc2.pdf]

Cell Reports

Article

Acute Iron Deprivation Reprograms Human Macrophage Metabolism and Reduces Inflammation *In Vivo*

Graphical Abstract

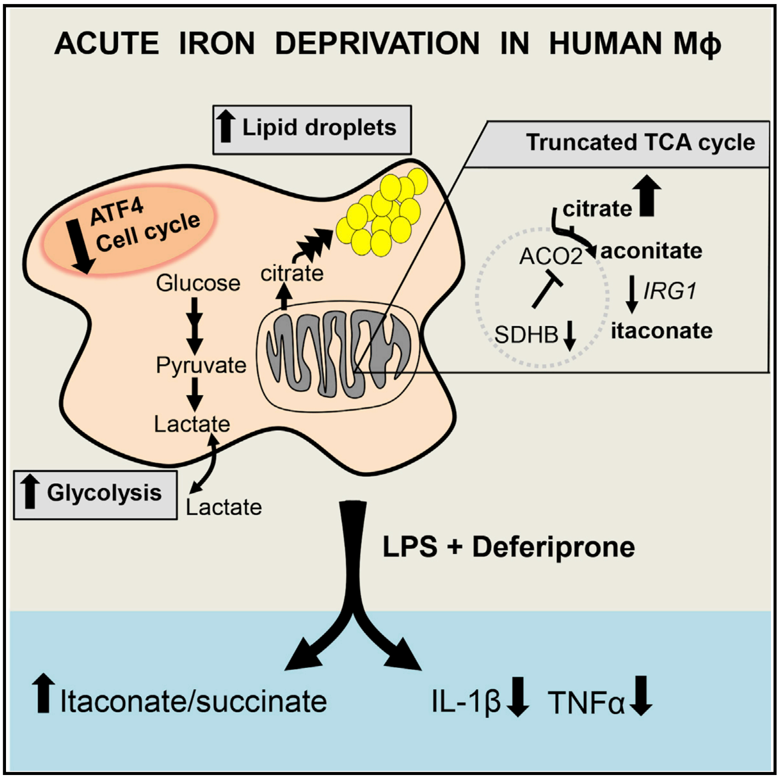

Highlights

- Acute iron deprivation causes an atypical Warburg effect in human macrophages
- Iron controls citrate and lipid pools and is essential for an intact TCA cycle
- LPS polarization is limited in iron-deprived human macrophages
- Acute iron deprivation reduces the severity of macrophage-driven glomerulonephritis

Authors

Marie Pereira, Tai-Di Chen, Norzawani Buang, ..., Stephen P. McAdoo, Christian Frezza, Jacques Behmoaras

Correspondence

jacques.behmoaras@imperial.ac.uk

In Brief

Iron is a crucial regulator of cell function, but its role in human macrophage immunometabolism is only partially understood. Pereira et al. show that acute iron deprivation in human macrophages causes anti-inflammatory immune and metabolic responses, and acute iron deprivation in rats reduces the severity of macrophage-driven renal inflammation.

Pereira et al., 2019, Cell Reports *28*, 498–511
July 9, 2019 © 2019 The Author(s).
https://doi.org/10.1016/j.celrep.2019.06.039

CellPress

OPEN ACCESS
CellPress

Cell Reports
Article

Acute Iron Deprivation Reprograms Human Macrophage Metabolism and Reduces Inflammation *In Vivo*

Marie Pereira,[1,4] Tai-Di Chen,[1,2,4] Norzawani Buang,[1] Antoni Olona,[1] Jeong-Hun Ko,[1] Maria Prendecki,[1] Ana S.H. Costa,[3] Efterpi Nikitopoulou,[3] Laura Tronci,[3] Charles D. Pusey,[1] H. Terence Cook,[1] Stephen P. McAdoo,[1] Christian Frezza,[3] and Jacques Behmoaras[1,5,*]
[1]Centre for Inflammatory Disease, Imperial College London, London W12 0NN, UK
[2]Department of Anatomic Pathology, Chang Gung Memorial Hospital, Taoyuan, Taiwan
[3]Medical Research Council Cancer Unit, University of Cambridge, Cambridge CB2 0XZ, UK
[4]These authors contributed equally
[5]Lead Contact
*Correspondence: jacques.behmoaras@imperial.ac.uk
https://doi.org/10.1016/j.celrep.2019.06.039

SUMMARY

Iron is an essential metal that fine-tunes the innate immune response by regulating macrophage function, but an integrative view of transcriptional and metabolic responses to iron perturbation in macrophages is lacking. Here, we induced acute iron chelation in primary human macrophages and measured their transcriptional and metabolic responses. Acute iron deprivation causes an anti-proliferative Warburg transcriptome, characterized by an ATF4-dependent signature. Iron-deprived human macrophages show an inhibition of oxidative phosphorylation and a concomitant increase in glycolysis, a large increase in glucose-derived citrate pools associated with lipid droplet accumulation, and modest levels of itaconate production. LPS polarization increases the itaconate:succinate ratio and decreases pro-inflammatory cytokine production. In rats, acute iron deprivation reduces the severity of macrophage-dependent crescentic glomerulonephritis by limiting glomerular cell proliferation and inducing lipid accumulation in the renal cortex. These results suggest that acute iron deprivation has *in vivo* protective effects mediated by an anti-inflammatory immunometabolic switch in macrophages.

INTRODUCTION

Iron, an essential metal for life, is a crucial regulator of innate immunity through macrophage function. Considering its vast pleiotropic roles in cellular homeostasis and its preferential enrichment in proteins belonging to mitochondria and the endoplasmic reticulum (ER; Andreini et al., 2018), iron has a master regulatory role in macrophage metabolism and its crosstalk with immune plasticity. Iron-dependent immunometabolism in macrophages occurs at multiple levels. Iron could be integrated into heme or iron-sulfur (Fe-S) clusters, which in turn are incorporated into specific protein families (hemoproteins and Fe-S cluster proteins) that orchestrate metabolic and immunological functions in macrophages (Aerbajinai et al., 2019; Drapier and Hibbs, 1988; Soares and Hamza, 2016; Tong et al., 2018). Furthermore, in its non-heme, Fe^{2+} form, iron can govern the activity of enzymes such as α-ketoglutarate (αKG)-dependent dioxygenases upstream of oxygen sensing and epigenetic modifications (Loenarz and Schofield, 2008; Nakazawa et al., 2016) or lipoxygenases responsible for generating bioactive lipid mediators (Haeggström and Funk, 2011). Besides its role in promoting immune activation in macrophages, iron is crucial in regulating the fundamental properties of eukaryotic cells such as DNA replication (Netz et al., 2011) and repair (Rudolf et al., 2006), and there is evidence of macrophage iron levels affecting the proliferation of stromal cells (Recalcati et al., 2019).

Macrophages show a sophisticated fine-tuning of intracellular iron availability during bacterial and fungal pathogenicity, a concept recently referred to as innate nutritional immunity (Andrianaki et al., 2018; Nairz et al., 2010; Núñez et al., 2018). Iron homeostasis affecting macrophage plasticity was also studied in the context of sterile inflammation and cancer. It has been reported that iron overloading in macrophages occurs in human chronic venous leg ulcers and cause an unrestrained pro-inflammatory M1-like phenotype (Sindrilaru et al., 2011). Likewise, increased intracellular iron polarizes the macrophages toward a detrimental pro-inflammatory state in the injured spinal cord (Kroner et al., 2014), and iron-positive microglia and macrophages in chronic active multiple sclerosis lesions are thought to be a source of inflammation that correlates with tissue damage and disease severity (Gillen et al., 2018). In keeping with these findings, in a mouse model of sickle disease, hemolysis and macrophage heme-iron accumulation trigger a proinflammatory phenoytpe in hepatic macrophages (Vinchi et al., 2016). In cancer, iron-loaded tumor-associated macrophages adopt a pro-inflammatory phenotype that can directly kill tumor cells (Costa da Silva et al., 2017), and the usage of iron oxide nanoparticles is an attractive avenue in cancer immunotherapy through the modulation of macrophage activity (Gu et al., 2019; Zanganeh et al., 2016). In summary, the findings from all of the

 © 2019 The Author(s).
This is an open access article under the CC BY license (http://creativecommons.org/licenses/by/4.0/).

OPEN ACCESS
CellPress

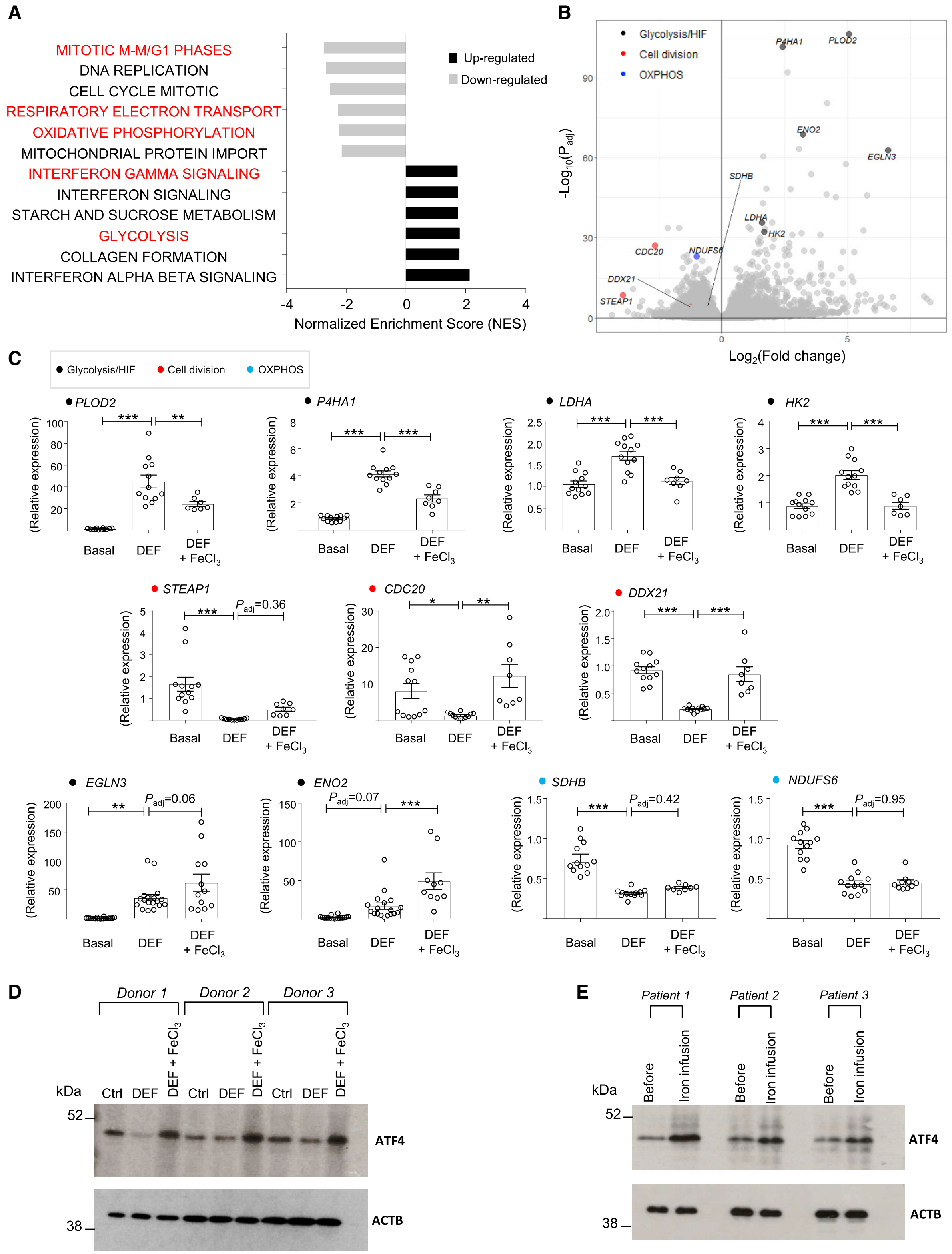

Figure 1. Acute Iron Deprivation Causes an Atypical Warburg Transcriptome and Mediates an ATF4-Driven Transcriptional Response in Human Macrophages

(A) Gene set enrichment analysis (GSEA) in upregulated and downregulated transcripts between control (basal) and DEF-treated human macrophages (500 μM, 24 h), measured by RNA-seq (n = 3 donors; Fc > 1.5; P_{adj} < 0.01).

(legend continued on next page)

above-mentioned studies converge toward a macrophage polarization profile resulting from prolonged exposure to iron in the tissue microenvironment, correlating with a pro-inflammatory (M1-like) phenotype. Although this phenotype associates with tissue damage during inflammatory disease (Kroner et al., 2014; Sindrilaru et al., 2011; Vinchi et al., 2016), it has been reported to be beneficial in cancer (Costa da Silva et al., 2017; Zanganeh et al., 2016).

Studies aiming to experimentally induce acute changes in cellular iron metabolism during inflammation are scarce. Reducing mitochondrial iron in the heart protects mice against ischemia-reperfusion injury (Chang et al., 2016). In human cell lines, acute iron deprivation results in profound mitochondria-driven metabolic changes, such as marked citrate accumulation followed by lipid synthesis and decreases the protein levels of several Fe-S cluster biogenesis proteins (Crooks et al., 2018; Tong et al., 2018). These studies suggest that acute manipulation of macrophage iron pools could reprogram its metabolism in the inflammatory microenvironment. However, a comprehensive study on the effects of iron manipulation in macrophage metabolism is lacking. Here, we induced acute iron chelation in primary human macrophages and measured their metabolic and transcriptional responses by stable isotope tracing and RNA sequencing (RNA-seq). We show that iron deficiency triggers hypoxia-inducible factor (HIF-1)-glycolysis and interferon (IFN) transcriptional responses, while there is a generalized downregulation of cell cycle-mitosis and oxidative phosphorylation (OXPHOS) pathways. We identify ATF4 as an iron-responsive transcription factor in human macrophages and show iron-dependent modulation of the expression of some of its known targets. These major responses were rescued with short exposure to $FeCl_3$ following iron chelation. Metabolically, glucose-derived citrate accumulates markedly and induces intracellular lipid droplet formation, and to a lesser extent itaconate synthesis, when macrophages lack iron. This is associated with the inhibition of the tricarboxylic acid (TCA) cycle, partly due to the downregulation of succinate dehydrogenase complex iron sulfur subunit B (SDHB) and the loss of mitochondrial aconitase activity. Consequently, OXPHOS is abolished and glycolysis is enhanced as a result of iron deprivation in human macrophages. Lipopolysaccharide (LPS) polarization of iron-deprived human macrophages shows that while the itaconate:succinate ratio is increased, the pro-inflammatory cytokines interleukin-1β (IL-1β) and tumor necrosis factor α (TNF-α) are reduced. This suggests that iron chelation restrains LPS polarization, which is further supported by RNA-seq analysis showing the induction of the transforming growth factor β (TGF-β) pathway in LPS-stimulated iron-deprived macrophages. *In vivo*, acute iron deprivation reduces the severity of macrophage-dependent crescentic glomerulonephritis by limiting glomerular cell proliferation and induces significant lipid droplet accumulation in the renal cortex.

Our study shows that acute iron deprivation results in a profound metabolic remodeling, promoting an atypical Warburg effect with anti-inflammatory properties in human macrophages. We confirm the beneficial effects of iron deprivation *in vivo* and propose macrophage iron manipulation as a potential therapeutic approach targeting immunometabolism in these cells.

RESULTS

Acute Iron Chelation Causes an Atypical Warburg Transcriptome in Human Macrophages

In mitochondria, the TCA cycle activity, respiration complexes, and heme biosynthesis are dependent on iron availability and Fe-S cluster biogenesis. Iron chelators with low membrane permeability such as deferoxamine cannot access the mitochondria, whereas deferiprone (DEF; 3-hydroxy-1,2-dimethylpyridin-4-one) is membrane permeable and can enter mitochondria (Sohn et al., 2008). RNA-seq of human macrophages treated with DEF showed a clear transcriptional response. As previously shown for iron chelation by chemical hypoxia mimetics (An et al., 1998; Peyssonnaux et al., 2007), we confirmed a glycolysis-HIF-P53 signature among the significantly upregulated gene families (Figures 1A and S1). These cells also show an induction of IFN signaling, while cell division-mitosis and OXPHOS were downregulated (Figure 1A). Given the previously established role of iron chelation in pseudohypoxia (Peyssonnaux et al., 2007) and cell division-mitosis (Le and Richardson, 2002), these results suggested iron-dependent transcriptomic changes in human macrophages. To further confirm that the transcriptional effects were due to the iron-chelation properties of DEF, human macrophages were treated with DEF, after which $FeCl_3$ was added for 8 h. qRT-PCR for the most significant genes belonging to glycolysis-HIF, OXPHOS, and cell division-mitosis showed significant rescue of the DEF effect with iron supplementation in some of the transcripts (Figures 1B and 1C). These results suggested that the major transcriptomics changes obtained with DEF treatment are due to iron deprivation in human macrophages. Since the results show that acute iron deprivation is associated with metabolic changes, we next examined how DEF compares to the well-established lipopolysaccharide (LPS)-mediated transcriptome characterized by increased glycolysis and attenuated OXPHOS in macrophages (Kelly and O'Neill, 2015; Mills et al., 2016). Comparative RNA-seq analysis between LPS and DEF-treated human macrophages showed that iron deprivation downregulated activating transcription factor 4 (ATF4)-mediated gene activation (Figure S1). This was not

(B) Volcano plot from RNA-seq highlighting genes belonging to glycolysis-HIF, cell division, and OXPHOS pathways.
(C) qRT-PCR for main glycolysis-HIF, cell division, and OXPHOS genes (color-coded) in iron-deprived (DEF, 500 μM, 24 h) and rescued human macrophages with short exposure (8 h) to 200 μM ferric chloride (DEF + $FeCl_3$); n = 4–9 donors/group.
(D) ATF4 western blotting in iron-deprived (DEF) and rescued (DEF + $FeCl_3$) human macrophages from three donors.
(E) ATF4 western blotting before and after 2 h of intravenous iron transfusion (Ferinject 1–1.5 g) in 3 CKD patients' PBMCs (see Method Details and Table S1 for patient information). *$p < 0.05$, **$p < 0.01$, and ***$p < 0.001$ by ANOVA followed by Tukey's multiple comparisons test. P_{adj}, adjusted p value; Fc, fold change. Error bars represent SEM.
See also Figures S1 and S2.

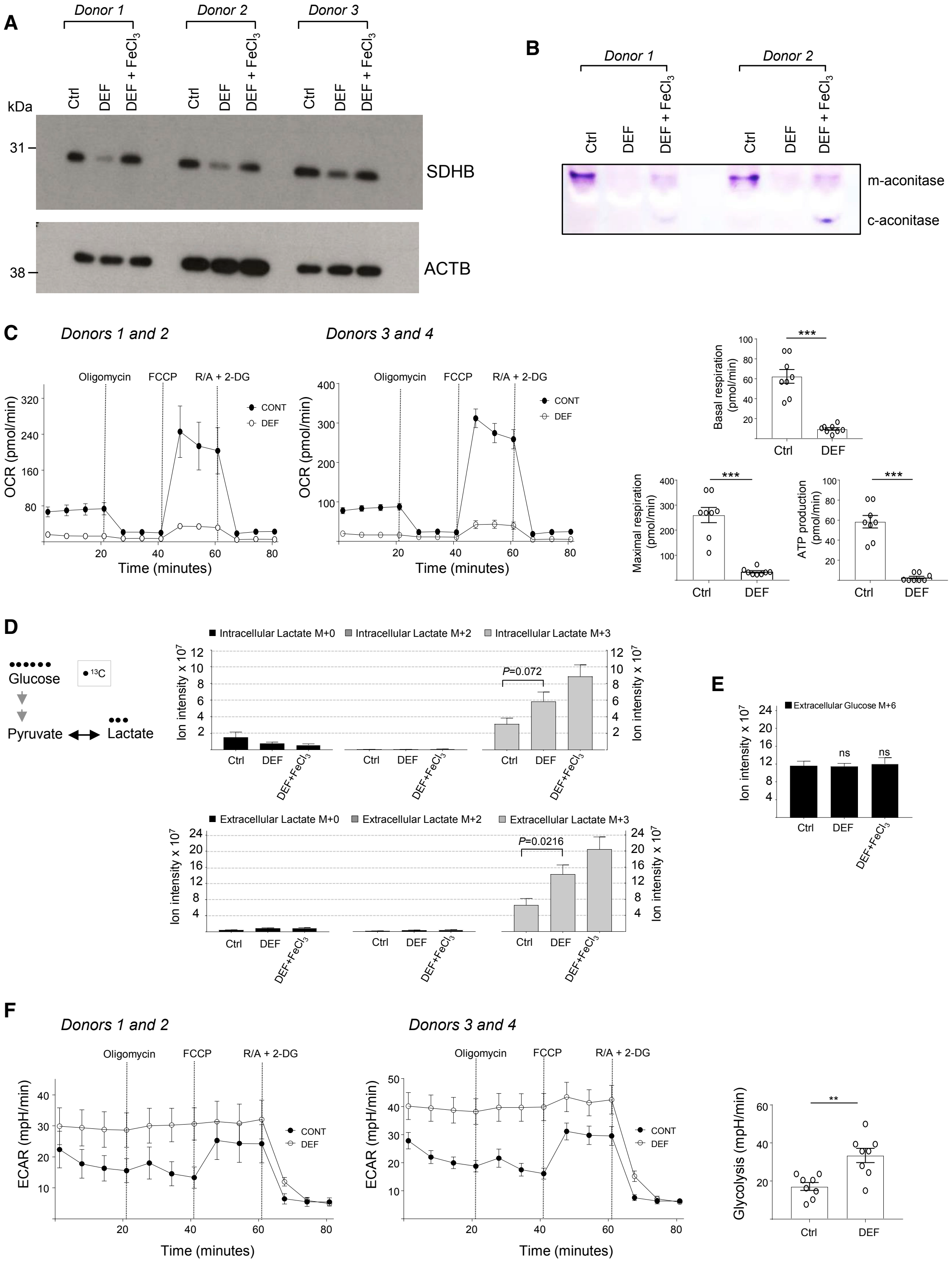

Figure 2. Acute Iron Deprivation Abolishes Oxidative Phosphorylation and Increases Aerobic Glycolytic Flux in Human Macrophages

(A) SDHB western blotting in iron-deprived (DEF) and rescued (DEF + $FeCl_3$) human macrophages from three donors. DEF (24 h, 500 μM); $FeCl_3$ (8 h, 200 μM).

(B) Aconitase activity gel in iron-deprived (DEF) and rescued (DEF + $FeCl_3$) human macrophages from two donors. m-aconitase, mitochondrial; c-aconitase, cytosolic.

(C) Oxygen consumption rate (OCR) measurement by extracellular flux analysis in control and DEF-treated human macrophages. Basal and maximal respiration and ATP production are shown at right. R/A, rotenone/antimycin; 2-DG, 2-deoxyglucose; DEF (24 h, 500 μM); n = 4 donors.

(legend continued on next page)

present in LPS-treated cells, which showed the upregulation of well-established Toll-like receptor, Janus kinase/signal transducer and activator of transcription (JAK/STAT), and nucleotide-binding oligomerization domain-like (NOD-like) receptor signaling pathways (Figure S1). We subsequently confirmed that ATF4 protein levels and the expression of some of its known target genes are dependent on acute changes in iron (Figures 1D and S1). To confirm the role of ATF4 in a more clinical setting, we examined the acute effects of intravenous iron administration in patients with stable, non-immune chronic kidney disease (CKD), who were receiving iron therapy as part of their routine clinical care. Peripheral blood mononuclear cells (PBMCs) were collected before and 120 min following intravenous iron (Ferinject 1–1.5 g) infusion, and ATF4 protein levels showed a clear increase following iron transfusion in all of the patients (Figure 1E), confirming its iron-mediated rapid induction.

Since metabolic consequences of acute iron deprivation and defective Fe-S cluster assembly are similar in human cell lines (Crooks et al., 2018), we next tested an alternative, drug-free method of acute iron deprivation and replenishment in human macrophages. RNA interference for *ISCU*, a primary Fe-S biogenesis scaffold protein known to have a master regulatory role in cellular iron levels, showed comparable transcriptomic responses to DEF (upregulation of glycolysis-HIF-IFN and downregulation of cell-cycle pathways) and the addition of $FeCl_3$ rescued the majority of the effects (Figure S2). Likewise, *ISCU* knockdown resulted in ATF4 downregulation, which was rescued with the addition of $FeCl_3$ in human macrophages (Figure S2).

Acute Iron Chelation Causes a Metabolic Switch in Human Macrophages

Iron deprivation resulted in increased glycolysis-HIF, decreased OXPHOS transcriptome pathways in human macrophages (Figure 1), indicative of a dysregulation of mitochondrial function. This was further supported by the suppression of the gene expression of respiratory chain enzymes containing Fe-S clusters, *NDUFS6* and *SDHB* (also known as the Fe-S subunit of complex II; Figure 1C). Since SDHB links the TCA cycle to OXPHOS (Murphy and O'Neill, 2018), we next investigated the iron-dependent control of its protein levels. SDHB protein levels were found to be controlled by intracellular iron levels, as iron replenishment following deprivation brought the levels of this protein back to its basal state (Figure 2A). As expected, iron-dependent mitochondrial aconitase activity was inhibited by DEF (Figure 2B). Iron replenishment partly restored mitochondrial aconitase activity and induced cytoplasmic aconitase activity in human macrophages (Figure 2B). In agreement with SDHB downregulation and loss of aconitase activity, OXPHOS was nearly totally inhibited following iron deprivation in human macrophages (Figure 2C). To measure the glycolytic rate, we incubated cells with uniformly labeled [U]-^{13}C-glucose and assessed the production of glucose-derived lactate using liquid chromatography-mass spectrometry (LC-MS). In line with the transcriptional changes, we found a significant increase in the secreted glucose-derived lactate (M+3) with no change in the consumption of glucose levels following DEF treatment (Figures 2D and 2E). Consistently, extracellular acidification rate (ECAR) was enhanced in DEF-treated human macrophages (Figure 2F). These results functionally validated the transcriptional analyses and confirmed that DEF elicits a metabolic switch whereby glycolysis is enhanced and mitochondrial function is suppressed.

A Citrate Checkpoint Characterizes Acute Iron Deprivation in Human Macrophages

To gain more insights into metabolic pathways affected by acute iron deprivation and replenishment, we incubated untreated, iron-deprived, and iron-repleted human macrophages with the tracer [U]-^{13}C-glucose and assessed its fate in the TCA cycle (Figure 3A). Iron deprivation and replenishment was well tolerated in human macrophages (Figure S3). We observed a striking increase in citrate levels upon DEF treatment (Figure 3B). The isotopologue distribution analysis (Figure 3A) revealed that the increased intracellular citrate pools were predominantly glucose derived (Figures 3B and S3). Glucose-derived citrate was partly converted to aconitate and itaconate (Figures 3A, 3B, and S3), but in relatively modest quantities, which is consistent with the inhibition of the iron-dependent mitochondrial aconitase activity (Figure 2B). Metabolites derived from the TCA cycle activity, such as M+2 glutamate and glutathione (GSH), were decreased, while M+2 malate remained unchanged (Figures S3 and S4). This was also the case for the extracellular glutamate, reflected by a reduction in M+2 glutamate (Figure S4). These results suggested an inhibition of the TCA cycle between aconitate and αKG. Furthermore, since nitric oxide synthases (NOSs) are heme-dependent enzymes responsible for nitric oxide generation from the oxidation of L-arginine to citrulline (Nathan and Xie, 1994), iron chelation showed an effect on cellular and extracellular arginine and citrulline pools in human macrophages (Figure S4).

Citrate is an important checkpoint metabolite in immunometabolism, critically positioned at the crosstalk between glucose catabolism, fatty acid synthesis, and oxidation (Tong and Rouault, 2007; Williams and O'Neill, 2018). We next hypothesized that marked citrate accumulation following acute iron deprivation could result in lipid droplet accumulation. In line with this hypothesis, DEF-treated human macrophages exhibited a striking increase in lipid droplet accumulation, which was diminished by co-incubation of DEF with $FeCl_3$ (Figure 3C).

(D) Intracellular and extracellular lactate (glucose-derived adduct shown at left) isotopologue quantification by LC-MS in iron-deprived (DEF, 500 μM, 24 h) and rescued human macrophages with short exposure to 200 μM ferric chloride (DEF + $FeCl_3$); n = 6 donors.

(E) Extracellular glucose M+6 by LC-MS; n = 6 donors.

(F) Extracellular acidification rate (ECAR) measured by extracellular flux analysis. R/A, rotenone/antimycin; 2-DG, 2-deoxyglucose, DEF (24 h, 500 μM); n = 4 donors.

Significance tested by t test (C, D, and F) and ANOVA followed by Dunnett's multiple comparisons test (E). ns, non-significant compared to Ctrl. $^{**}p < 0.01$ and $^{***}p < 0.001$. Error bars represent SEM.

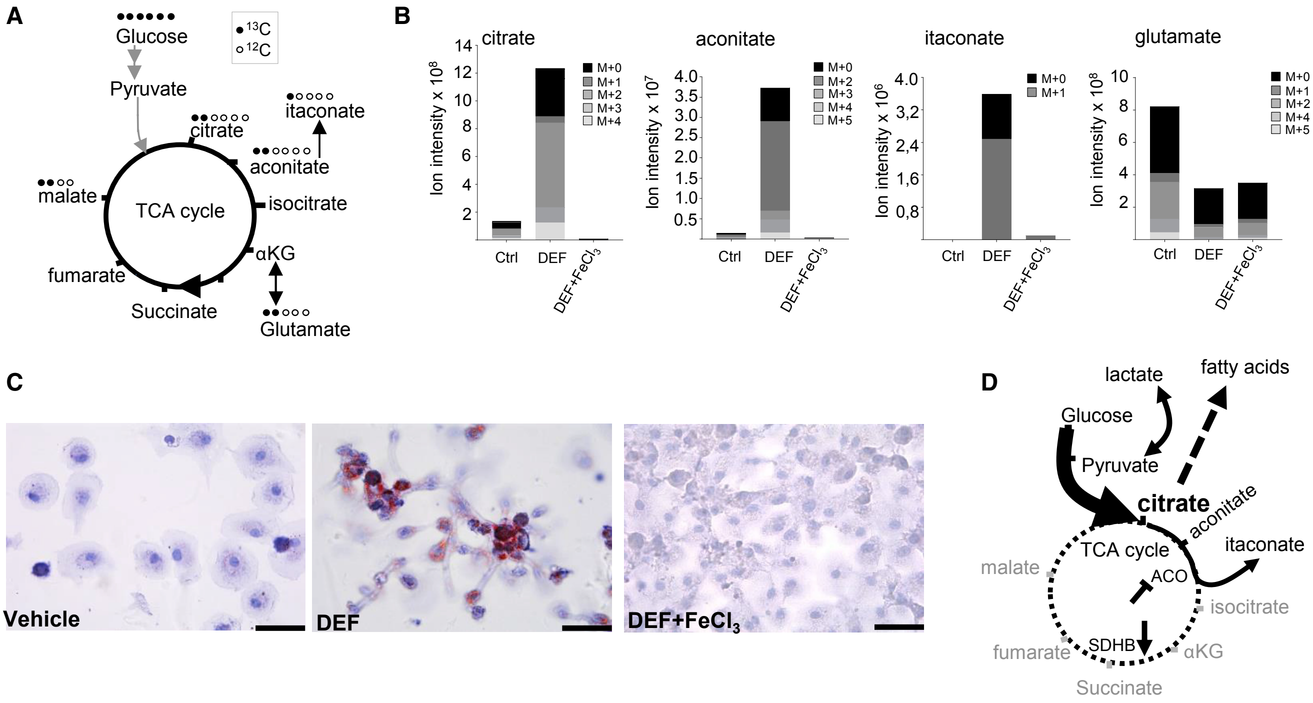

Figure 3. A Citrate Checkpoint Characterizes Iron-Deprived Human Macrophages

(A) Diagram of uniformly labeled [U]-^{13}C-glucose catabolism, highlighting the TCA cycle metabolites and their expected glucose-derived ^{13}C atoms.

(B) Stacked isotopologues measured by LC-MS shown for citrate, aconitate, itaconate, and glutamate in iron-deprived (DEF, 500 μM, 24 h) and rescued human macrophages with short exposure (8 h) to 200 μM ferric chloride (DEF + $FeCl_3$); n = 6 donors.

(C) Vehicle, DEF-treated, or concomitant DEF and $FeCl_3$-treated human macrophages stained for oil red O. The results are representative of n = 4 donors.

(D) Summary of the TCA cycle changes due to iron deprivation in human macrophages. Citrate accumulation resulting from aconitase (ACO) inhibition and SDHB downregulation and the fate of citrate-derived metabolites are illustrated. Dashed circle represents inhibited TCA cycle activity. αKG, α-ketoglutarate.

Scale bars, 40 μm.

See also Figures S3 and S4.

Lipid accumulation was similarly reduced when the fatty acid synthase (FASN) inhibitor cerulenin was used together with DEF treatment (Figure S4), suggesting that *de novo* synthesis is engaged in iron-depleted macrophages.

In summary, the metabolic reprogramming due to iron deprivation results in marked citrate accumulation, which partly translates into aconitate and itaconate production together with lipid droplet accumulation (Figure 3D). Oxidative phosphorylation is inhibited and αKG-derived metabolites show a decrease, suggesting overall diminished activity of the TCA cycle. This causes a compensatory increase in aerobic glycolysis with the subsequent production and secretion of lactate (Figure 3D).

Acute Iron Deprivation and Pro-inflammatory Macrophage Polarization

To find out how this specific iron-deficient macrophage phenotype undergoes the classical pro-inflammatory activation, we polarized DEF-treated human macrophages with LPS stimulation and measured IL-1β and TNF-α production. The results showed a reduction in secreted factors in iron-chelated macrophages (Figure 4A). We then performed a time course analysis of LPS treatment in DEF-treated macrophages and measured 88 metabolites by LC-MS, including citrate, aconitate, itaconate, and succinate (Figure 5). We first confirmed the truncated TCA cycle in LPS-stimulated iron-deprived macrophages with increased citrate (and citrate:succinate ratio) and aconitate and decreased αKG and glutamate levels (Figures 4B, 4C, and S5). Itaconate production is further augmented in DEF-treated human macrophages, and the itaconate:succinate ratio was increased at 24 h (Figures 4B and 4C), which is indicative of overall anti-inflammatory metabolic switching (Murphy and O'Neill, 2018). *IRG1* mRNA levels did not show an apparent DEF effect, suggesting that the effects of iron deprivation on itaconate levels are likely to be mediated by changes in metabolic fluxes, rather than by the transcriptional regulation of *IRG1* (Figure S5). Comparative RNA-seq analysis between LPS and LPS + DEF-treated macrophages during early activation (3 h) confirmed the results obtained with Ctrl versus DEF comparison (i.e., increased glycolysis, IFN-γ signaling, decreased OXPHOS, and DNA replication; Figure S5). Iron-depleted macrophages stimulated with LPS showed upregulation of the TGF-β signaling pathway, which is associated with increases in *TGFB1*, *VEGFA*, *CTGF*, *CXCL12*, *IL10*, and *IL1RN* expression levels (Figure S5). Considering the anti-inflammatory role of itaconate (Bambouskova et al., 2018; Mills et al., 2018), these results suggest that acute iron chelation limits the classical macrophage polarization by increasing the itaconate:succinate ratio, decreasing secreted IL-1β, and TNF-α, and promoting the early induction of the TGF-β signaling pathway.

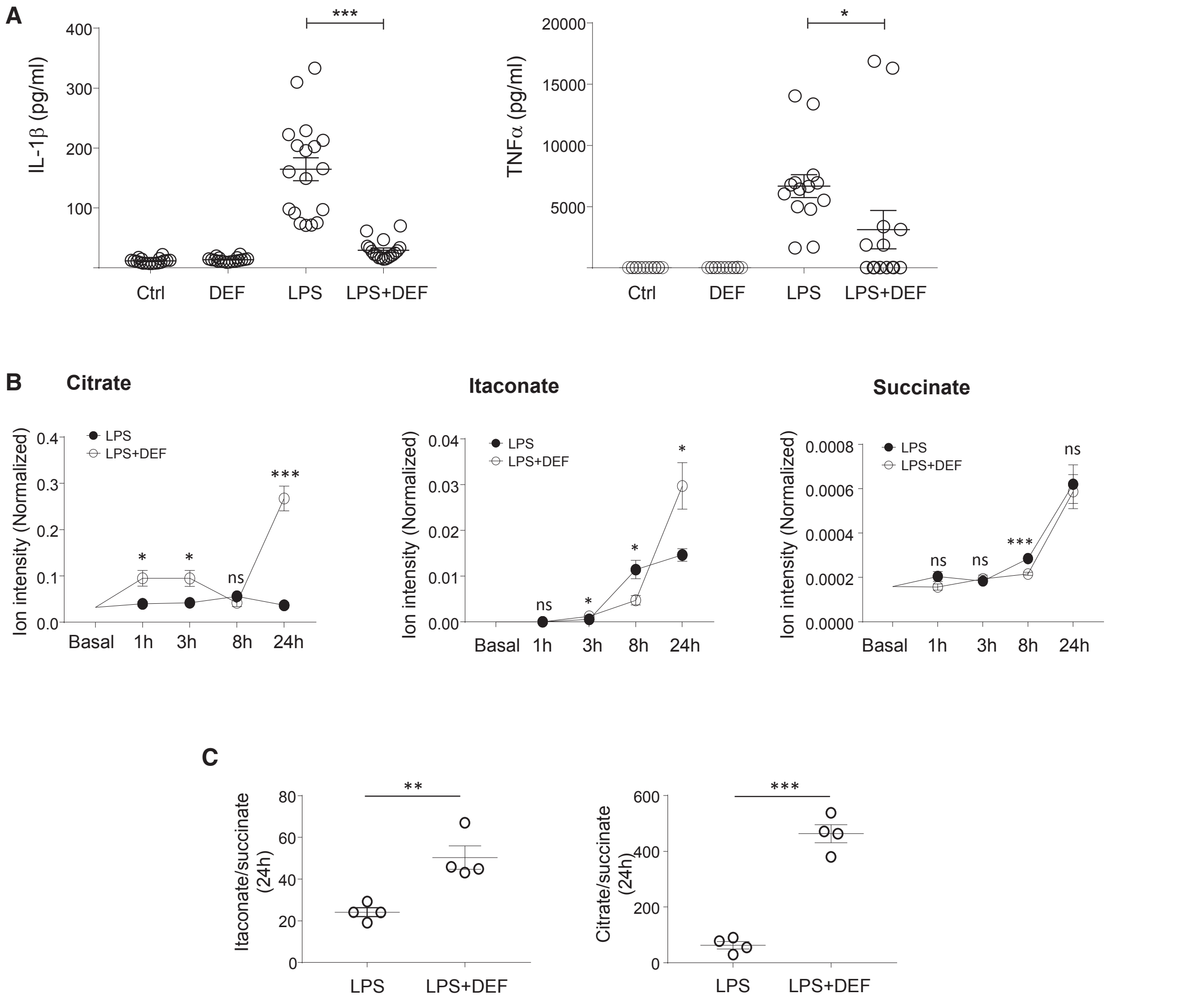

Figure 4. Acute Iron Chelation Limits the Pro-inflammatory Macrophage Polarization

(A) IL-1β and TNF-α production in iron-deprived human macrophages (500 μM, 24 h), following LPS treatment (100 ng/mL, 24 h; LPS + DEF); n = 7 donors.

(B) LC-MS for citrate, itaconate, and succinate in basal and DEF-treated macrophages (500 μM, 24 h) throughout an LPS (100 ng/mL) time course; n = 4 donors.

(C) Itaconate:succinate and citrate:succinate ratios at 24 h; n = 4 donors.

Significance tested by ANOVA followed by Tukey's multiple comparisons test (A) and t test (B and C). *p < 0.05, **p < 0.01, and ***p < 0.001. Error bars represent SEM.

See also Figure S5.

Acute Iron Deprivation Is Preventive in Glomerulonephritis and Induces *De Novo* Lipogenesis

To investigate the pathophysiological significance of acute iron deprivation in a macrophage-dependent inflammatory disease model, crescentic glomerulonephritis was induced in the Wistar Kyoto rats treated either with vehicle or DEF (Figure 6A). Oral DEF treatment markedly lowered the levels of proteinuria during the entire disease course (Figure 6B) and ameliorated glomerular morphology (Figure 6C). The glomeruli of the DEF-treated group had a substantially reduced fibrin score and crescent formation as well as decreased glomerular size (Figure 6D). Since the WKY nephrotoxic nephritis (NTN) model depends on the infiltration and activation of monocytes and macrophages (Behmoaras et al., 2010), we performed ED-1 (rat CD68) staining and showed that DEF significantly decreased the number of monocytes and macrophages in the glomeruli (Figure S6). We then checked whether the lipid accumulation as a result of iron deprivation was conserved *in vivo* and found increased oil red O staining in DEF-treated rat glomeruli and renal cortex following NTN (Figure 6E), suggesting that the citrate-derived *de novo* fatty acid synthesis occurs during acute iron deprivation in experimental crescentic glomerulonephritis. Furthermore, since iron deprivation caused a downregulation of cell cycle-mitosis pathways measured by RNA-seq (Figure 1A), we measured intraglomerular cell proliferation and found decreased proliferating glomerular cell nuclear antigen (PCNA) in DEF-treated animals following the induction of NTN (Figure 6F).

To assess whether DEF has therapeutic effects after the onset of glomerulonephritis, rats were treated with or without DEF from day 4 following the induction of NTN, which corresponds to the

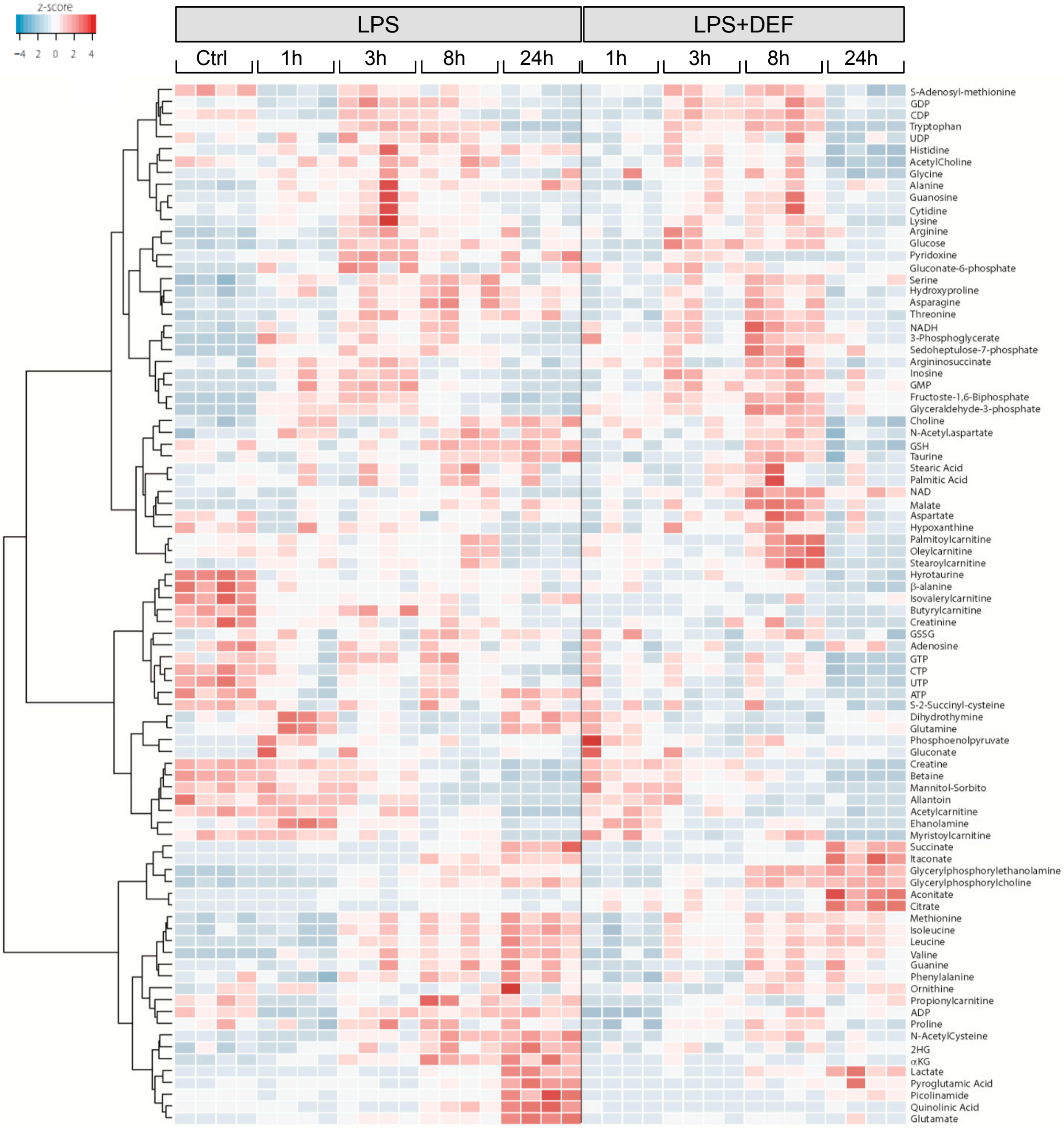

Figure 5. Acute Iron Chelation and Time Course Metabolomics in LPS-Stimulated Human Macrophages

Heatmap for LC-MS analysis of 88 metabolites in basal (Ctrl), LPS treated (1, 3, 8, and 24 h) and LPS + DEF-treated human macrophages; DEF treatment is 500 μM, 24 h; n = 4 donors.

onset of renal damage and proteinuria. The rats were then sacrificed on day 10, at the peak of glomerular inflammation (Figure 6G). Notably, the progression of proteinuria at day 10 was reversed in rats treated with DEF when compared to either the treated group on day 7 or their control counterparts at day 10 (Figure 6H). The glomerular morphology of DEF-treated rats at day 10 showed improved features when compared to vehicle-treated rats (Figure 6I), which was also accompanied by reduced fibrin score and crescent formation together with decreased glomerular size (Figure 6J). Monocyte/macrophage infiltration was significantly decreased by DEF (Figure S6), and acute iron deprivation by DEF resulted in ameliorated renal function (i.e., reduced creatinine and blood urea nitrogen) associated with reduced intraglomerular cell proliferation (Figure S6). DEF treatment did not alter erythropoiesis as hemoglobin levels were not significantly altered (Figure S6). In addition to its therapeutic effects *in vivo*, DEF reduced superoxide production in the NTN-susceptible WKY rat bone marrow-derived macrophages (BMDMs) and its antioxidant properties were confirmed in a cell-free superoxide production system (Figure S6). In summary, *in vivo* iron chelation in macrophage-dependent renal inflammation is accompanied by metabolic and transcriptomic changes (*de novo* lipid accumulation, anti-proliferative responses) that are conserved *in vitro* cultured human macrophages.

OPEN
ACCESS
CellPress

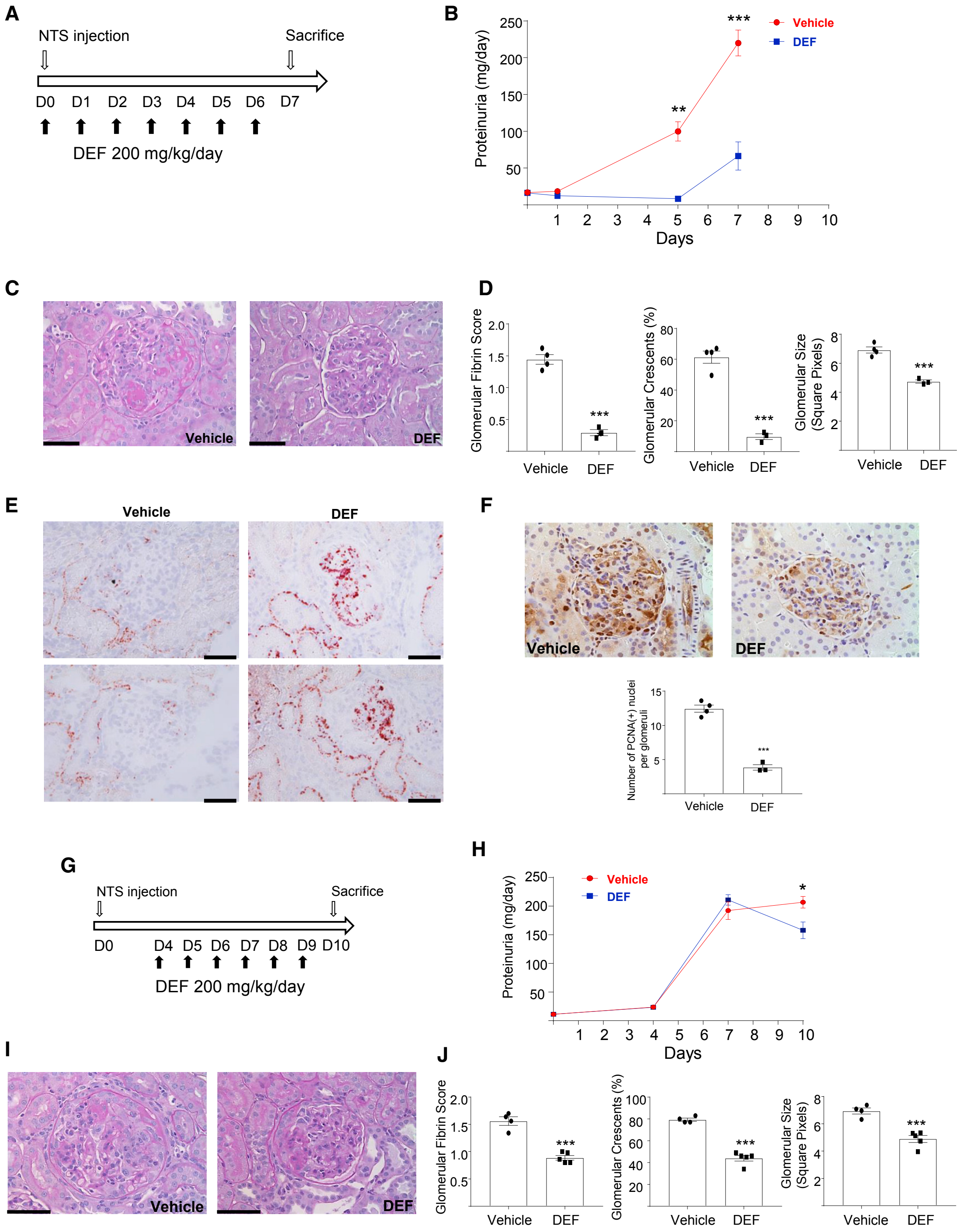

Figure 6. Acute Iron Deprivation Protects against Crescentic Glomerulonephritis and Induces *De Novo* Lipid Accumulation

(A) Experimental design.

(B) Amount of urinary protein at different time points during the course of NTN; n=3 or 4 rats/group.

(C) PAS staining showing representative glomerular morphology of the vehicle (left) and the DEF (right) groups.

(D) Glomerular fibrin score, percentage of glomeruli with crescents, and glomerular size in vehicle and DEF-treated rats; n = 3 or 4 rats/group.

(E) Nephritic glomeruli of vehicle- or DEF-treated rats showing oil red O^+ glomerular and tubular cells in the DEF group.

(F) PCNA staining showing proliferating cells in nephritic glomeruli; number of nuclear PCNA(+) cells in the glomeruli (bottom); n = 3 or 4 rats/group.

(G) Experimental design for the therapeutic experiment.

(legend continued on next page)

DISCUSSION

Iron serves as a prosthetic group for proteins orchestrating immunometabolic responses at multiple levels in macrophages. For instance, mammalian complex I hosts as many as eight Fe-S clusters (Vinothkumar et al., 2014), allowing optimal electron transfer during OXPHOS and maintaining the balance between the generation of reactive oxygen species (ROS) and ATP production (Mills et al., 2016). Furthermore, non-mitochondrial ROS production, an essential feature of macrophage-driven immune phagocytic responses, is partly mediated by NOX2 (CYBB), which uses heme-iron for superoxide generation (Sumimoto, 2008). In this study, we showed that iron deprivation caused an induction of glycolysis-HIF and a repression of OXPHOS pathways in human macrophages. Dendritic cells and macrophages, when stimulated with pro-inflammatory stimuli, decrease OXPHOS with a simultaneous upregulation of glycolysis and activity in the pentose phosphate pathway (Krawczyk et al., 2010; Mills et al., 2016; Tannahill et al., 2013). Although iron deprivation and pro-inflammatory M1-type macrophage stimulation share the same metabolic switch, the underlying mechanisms are likely to differ. The inhibition of OXPHOS is explained by nitric oxide-mediated nitrosylation of mitochondrial Fe-S clusters in LPS-stimulated murine macrophages (Drapier and Hibbs, 1988; Everts et al., 2012). Instead, iron deprivation directly affects the expression and protein levels of respiratory chain Fe-S enzymes such as *NDUFS6* and *SDHB*. It causes the loss of OXPHOS and ATP production, partly by reducing SDHB levels in conjunction with the inhibition of mitochondrial aconitase activity. The rapid increase in SDHB levels following iron replenishment suggests the existence of an iron-responsive element in *SDHB* mRNA, which was previously reported in *Drosophila* (Kohler et al., 1995). Furthermore, SDHB deletion was associated with reduced IL-1β and HIF-1α in LPS-stimulated macrophages (Mills et al., 2016), although LPS stimulation itself was shown to diminish SDHB protein levels in RAW264.7 cells (Tong et al., 2018). Considering the large-scale suppression of Fe-S cluster biogenesis proteins in response to TLR4 activation in murine cell lines (Tong et al., 2018) and the previously described iron-sequestration phenotype in M1 human macrophages (Recalcati et al., 2010), one could speculate that mitochondrial iron deprivation, M1 activation, and ISCU small interfering RNA (siRNA) or mutagenesis share in the common inhibition of OXPHOS and a concomitant increase in aerobic glycolysis in macrophages.

DEF is an orally active Food and Drug Administration (FDA)-approved iron chelator that preferentially binds to ferric iron (Fe^{3+}) in a 3:1 configuration and is used as alternative or complementary to deferoxamine treatment in hemoglobinopathies characterized by iron overload (Hider and Hoffbrand, 2018). DEF can enter the mitochondria and shuttle iron between subcellular compartments (Sohn et al., 2008). Because of its low affinity to iron, DEF is less likely to cause iron depletion, which we also confirmed by measuring DEF-treated mitochondrial and cytosolic iron in human macrophages (data not shown). The ability of DEF to chelate mitochondrial iron and to make it available for other cellular compartments (Kakhlon et al., 2008) has therapeutic benefit for disorders such as Friedreich ataxia characterized by altered mitochondrial Fe-S cluster biogenesis (Pandolfo and Hausmann, 2013). More generally, mitochondrial iron handling is central to regulating cellular and systemic homeostasis (Paul et al., 2017). In this context, the distinct properties of DEF make this drug ideal for studying the metabolic consequences arising from mitochondrial iron imbalances rather than measuring the indirect effects of extracellular iron chelation obtained by using deferoxamine. Iron imbalances between mitochondria and cytosol can have broad pathological consequences in cancer through altered immunometabolism (Li et al., 2018), and the genetic deficiencies of mitochondria-related genes (*FH*, *PINK1*) are likely to trigger dysregulation of the mitochondria-cytosol iron balance, which is associated with the Warburg effect and HIF signature in cancer (Li et al., 2018; Tong et al., 2011; Tyrakis et al., 2017). We observed a macrophage pseudohypoxia transcriptome (i.e., HIF response in the presence of oxygen; Selak et al., 2005; Tannahill et al., 2013) with DEF treatment, which was previously described in LPS-stimulated macrophages (Tannahill et al., 2013). One limitation of our study is that the use of $FeCl_3$ for iron replenishment *in vitro* does not mimic exposure to heme-iron, which occurs *in vivo* through the phagocytosis of red blood cells by macrophages due to hemorrhage, as reported in spinal cord injury (Kroner et al., 2014). In hemolytic diseases, which are characterized by the enhanced release of hemoglobin and heme into the circulation, heme-iron loading of reticulo-endothelial system macrophages changes their activation (Vinchi et al., 2016). Nonetheless, macrophages can also be exposed to non-heme iron released from dying cells, and it should also be noted that the pro-oxidant properties $FeCl_3$ may exacerbate the oxidative stress and trigger the ATF4 response.

One major transcriptional response that was specific to DEF treatment was ATF4 downregulation. Given the induction of this transcription factor in oxidative and ER stress responses (Ameri and Harris, 2008) and during heme-dependent erythroid differentiation (Suragani et al., 2012), this result confirms the antioxidant properties of DEF and its effect on superoxide neutralization. Furthermore, ATF4 reprograms $CD4^+$ T cell metabolism and immune response (Yang et al., 2018), and it is therefore conceivable that it has a similar role in human macrophages, especially given its effect on glutathione levels (Yang et al., 2018). Another distinct feature of acute iron deprivation was its modest itaconate production when compared to LPS-stimulated human macrophages, which produce large amounts of this anti-inflammatory metabolite (Mills et al., 2018; Papathanassiu et al., 2017). The limited itaconate production with iron deprivation

(H) Amount of urinary protein levels at different time points during the course of NTN; n = 4 or 5 rats/group.
(I) PAS staining showing representative glomerular morphology of the vehicle (left) and DEF (right) groups.
(J) Glomerular fibrin score, percentage of glomeruli with crescents, and glomerular size in vehicle- and DEF-treated rats; n = 4 or 5 rats/group.
*p < 0.05, **p < 0.01, and ***p < 0.001 by t test. Scale bars, 40 μm. Error bars represent SEM.
See also Figure S6.

could come from the type 1 interferon response affecting IRG1 gene expression (Mills et al., 2018). Considering the anti-inflammatory (Lampropoulou et al., 2016; Mills et al., 2018) and pro-tumorigenic (Weiss et al., 2018) effects of itaconate, it may contribute to the broadly anti-inflammatory macrophage phenotype we observe following iron deprivation. LPS stimulation augments itaconate production in iron-deprived macrophages and increases the itaconate:succinate ratio, while decreasing IL-1β and TNF-α secretion and promoting the TGF-β signaling pathway. These results indicate that acute iron chelation limits classic LPS polarization partly through the immunomodulatory properties of itaconate. A recent addition to the latter is the inhibitory effects of itaconate during immune tolerance (Dominguez-Andres et al., 2019). Furthermore, itaconate is bactericidal, which is in line with the previously established antibacterial properties of iron chelators (Thompson et al., 2012).

Acute iron deprivation in human macrophages resulted in a robust citrate accumulation, which could be primarily attributed to aconitase inhibition. Mitochondrial and cytosolic aconitases catalyze the interconversion of citrate and isocitrate, and their activities are affected by cellular iron and oxidative levels and the activity of Fe-S biogenesis (Tong and Rouault, 2007). Citrate can be transported across the inner mitochondrial membrane via the tricarboxylate carrier, and once in the cytosol, it is the substrate of ATP-citrate lyase, which generates acetyl-coenzyme A (CoA), the building block of cholesterol and the fatty acids. *De novo* fatty acid synthesis is the hallmark of LPS-stimulated dendritic cells (Everts et al., 2014) and macrophages (Carroll et al., 2018). Fatty acid oxidation has been associated with pro-inflammatory macrophage activation (Hall et al., 2013, 2018), which highlights the complexity of lipid metabolism and the possible master-regulatory role of citrate in fine-tuning context-dependent macrophage responses. Here, we found that macrophages accumulate lipid droplets as a result of iron deprivation. In contrast with what was observed with TNF/IFN stimulation (Infantino et al., 2014), the marked citrate accumulation was not associated with a pro-inflammatory signal, suggesting that citrate pools are preferentially used for lipogenesis, causing steatosis in macrophages and nephritic kidneys. Citrate and iron metabolism are intimately linked as citrate can chelate divalent cations such as Fe^{2+} and is proposed to be one of the carriers of non-transferrin-bound iron (Grootveld et al., 1989). It can also inhibit succinate oxidation (Hillar et al., 1975). Systemically, elevated citrate levels have been linked to increased hepcidin mRNA expression in primary hepatocytes and *in vivo* (da Silva et al., 2017), suggesting the existence of a citrate-iron regulatory feedback loop in macrophages. Citrate has also been linked to IFN-γ production through the acetylation of glyceraldehyde 3-phosphate dehydrogenase (GAPDH) in T cells (Balmer et al., 2016), a mechanism that could explain enhanced IFN response in iron-deprived human macrophages.

Studies on macrophage polarization following the manipulation of intracellular iron levels by different means has resulted in contrasting results, presumably because of the different model systems and the possible existence of a critical concentration of intracellular iron that likely drives the polarization (Recalcati et al., 2019). While treatment with hepcidin resulted in the attenuation of the inflammatory response in LPS-treated macrophages (De Domenico et al., 2010), dietary iron restriction rendered spleen macrophages pro-inflammatory following sublethal LPS injection in mice (Pagani et al., 2011). No obvious changes in BMDM polarization markers were observed following the conditional deletion of ferroportin from the myeloid lineage (Recalcati et al., 2019). More recently, and in accordance with our findings, the knock down of glia maturation factor-γ (GMFG) in macrophages was shown to cause an iron-deficiency response and M2 macrophage polarization associated with reduced mitochondrial respiration chain components, ISCU, and basal oxygen consumption and an increased HIF response (Aerbajinai et al., 2019). Likewise, treatment with iron led to M1 polarization in mice BMDMs (Handa et al., 2019).

The acute iron chelation in experimental glomerulonephritis resulted in *de novo* lipid synthesis but ameliorated the renal outcome. Considering that defective fatty acid oxidation was linked to fibrosis in CKD (Kang et al., 2015), these results argue in favor of a possible transient beneficial role of *de novo* lipogenesis in inflammation, as observed by others (Papazyan et al., 2016; Solinas et al., 2015). The systemic use of DEF in macrophage-dependent inflammatory disease should take into account the extent of iron loading in macrophages residing in different tissues. In fact, depending on tissue-dependent variation in ferroportin levels (Drakesmith et al., 2015), the macrophage iron content is likely to differ in different organs, and systemic iron deprivation may not have the same effect on infiltrating and tissue-resident macrophages.

When compared with the well-known LPS/Toll-like receptor 4 (TLR4)-induced Warburg effect in macrophages, iron deprivation-mediated immunometabolism could be considered atypical (i.e., a sterile Warburg effect devoid of pro-inflammatory programming), suggesting the different degrees of this phenomenon in the spectrum model of macrophage plasticity. In summary, immunometabolic changes derived from acute iron deprivation in macrophages could be considered a future therapeutic angle in macrophage-dependent inflammatory disease.

STAR★METHODS

Detailed methods are provided in the online version of this paper and include the following:

- KEY RESOURCES TABLE
- LEAD CONTACT AND MATERIALS AVAILABILITY
- EXPERIMENTAL MODEL AND SUBJECT DETAILS
  - Rats
  - CKD patients
- METHOD DETAILS
  - Isolation of macrophages and PBMCs
  - Metabolic extracellular flux analysis
  - Stable isotope tracing by liquid chromatography-mass spectrometry (LC-MS)
  - RNA extraction and library preparation
  - Bioinformatics
  - Quantitative RT-PCR
  - Western blotting
  - RNA interference
  - ELISA

- ○ Nephrotoxic Nephritis and immunohistochemistry
- ○ Oil-red-o staining
- ○ Aconitase in-gel assay
- QUANTIFICATION AND STATISTICAL ANALYSIS
- DATA AND CODE AVAILABILITY

SUPPLEMENTAL INFORMATION

Supplemental Information can be found online at https://doi.org/10.1016/j.celrep.2019.06.039.

ACKNOWLEDGMENTS

This work was supported by the Medical Research Council (MR/M004716/1 and MR/N01121X/1, to J.B., and MRC_MC_UU_12022/6, to C.F.). We are grateful to Dr. Wing-Hang Tong (National Institute of Child Health and Human Development) for providing guidance on in-gel aconitase assays. We thank Hal Drakesmith for providing critical feedback on the manuscript.

AUTHOR CONTRIBUTIONS

Conceptualization, J.B.; Methodology, J.B. and T.-D.C.; Investigation, M. Pereira, T.-D.C., N.B., A.O., M. Prendecki, J.-H.K., A.S.H.C., E.N., L.T., C.D.P., H.T.C., S.P.M., C.F., and J.B.; Writing – Original Draft, J.B.; Writing – Review & Editing, J.B., T.-D.C., and C.F.; Visualization, J.B., T.-D.C., and C.F.; Supervision, J.B. and C.F.; Funding Acquisition, J.B. and C.F. All of the authors read and approved the final manuscript.

DECLARATION OF INTERESTS

The authors declare no competing interests.

Received: December 11, 2018
Revised: April 29, 2019
Accepted: June 7, 2019
Published: July 9, 2019

REFERENCES

Aerbajinai, W., Ghosh, M.C., Liu, J., Kumkhaek, C., Zhu, J., Chin, K., Rouault, T.A., and Rodgers, G.P. (2019). Glia maturation factor-γ regulates murine macrophage iron metabolism and M2 polarization through mitochondrial ROS. Blood Adv. *3*, 1211–1225.

Ameri, K., and Harris, A.L. (2008). Activating transcription factor 4. Int. J. Biochem. Cell Biol. *40*, 14–21.

An, W.G., Kanekal, M., Simon, M.C., Maltepe, E., Blagosklonny, M.V., and Neckers, L.M. (1998). Stabilization of wild-type p53 by hypoxia-inducible factor 1alpha. Nature *392*, 405–408.

Andreini, C., Putignano, V., Rosato, A., and Banci, L. (2018). The human iron-proteome. Metallomics *10*, 1223–1231.

Andrianaki, A.M., Kyrmizi, I., Thanopoulou, K., Baldin, C., Drakos, E., Soliman, S.S.M., Shetty, A.C., McCracken, C., Akoumianaki, T., Stylianou, K., et al. (2018). Iron restriction inside macrophages regulates pulmonary host defense against Rhizopus species. Nat. Commun. *9*, 3333.

Balmer, M.L., Ma, E.H., Bantug, G.R., Grählert, J., Pfister, S., Glatter, T., Jauch, A., Dimeloe, S., Slack, E., Dehio, P., et al. (2016). Memory CD8(+) T Cells Require Increased Concentrations of Acetate Induced by Stress for Optimal Function. Immunity *44*, 1312–1324.

Bambouskova, M., Gorvel, L., Lampropoulou, V., Sergushichev, A., Loginicheva, E., Johnson, K., Korenfeld, D., Mathyer, M.E., Kim, H., Huang, L.H., et al. (2018). Electrophilic properties of itaconate and derivatives regulate the IκBζ-ATF3 inflammatory axis. Nature *556*, 501–504.

Behmoaras, J., Smith, J., D'Souza, Z., Bhangal, G., Chawanasuntoropoj, R., Tam, F.W., Pusey, C.D., Aitman, T.J., and Cook, H.T. (2010). Genetic loci modulate macrophage activity and glomerular damage in experimental glomerulonephritis. J. Am. Soc. Nephrol. *21*, 1136–1144.

Carroll, R.G., Zasłona, Z., Galván-Peña, S., Koppe, E.L., Sévin, D.C., Angiari, S., Triantafilou, M., Triantafilou, K., Modis, L.K., and O'Neill, L.A. (2018). An unexpected link between fatty acid synthase and cholesterol synthesis in proinflammatory macrophage activation. J. Biol. Chem. *293*, 5509–5521.

Chang, H.C., Wu, R., Shang, M., Sato, T., Chen, C., Shapiro, J.S., Liu, T., Thakur, A., Sawicki, K.T., Prasad, S.V., and Ardehali, H. (2016). Reduction in mitochondrial iron alleviates cardiac damage during injury. EMBO Mol. Med. *8*, 247–267.

Costa da Silva, M., Breckwoldt, M.O., Vinchi, F., Correia, M.P., Stojanovic, A., Thielmann, C.M., Meister, M., Muley, T., Warth, A., Platten, M., et al. (2017). Iron Induces Anti-tumor Activity in Tumor-Associated Macrophages. Front. Immunol. *8*, 1479.

Crooks, D.R., Maio, N., Lane, A.N., Jarnik, M., Higashi, R.M., Haller, R.G., Yang, Y., Fan, T.W., Linehan, W.M., and Rouault, T.A. (2018). Acute loss of iron-sulfur clusters results in metabolic reprogramming and generation of lipid droplets in mammalian cells. J. Biol. Chem. *293*, 8297–8311.

da Silva, A.R., Neves, J., Mleczko-Sanecka, K., Tandon, A., Sauer, S.W., Hentze, M.W., and Muckenthaler, M.U. (2017). Cellular citrate levels establish a regulatory link between energy metabolism and the hepatic iron hormone hepcidin. J. Mol. Med. (Berl.) *95*, 851–860.

De Domenico, I., Zhang, T.Y., Koening, C.L., Branch, R.W., London, N., Lo, E., Daynes, R.A., Kushner, J.P., Li, D., Ward, D.M., and Kaplan, J. (2010). Hepcidin mediates transcriptional changes that modulate acute cytokine-induced inflammatory responses in mice. J. Clin. Invest. *120*, 2395–2405.

Dominguez-Andres, J., Novakovic, B., Li, Y., Scicluna, B.P., Gresnigt, M.S., Arts, R.J.W., Oosting, M., Moorlag, S., Groh, L.A., Zwaag, J., et al. (2019). The Itaconate Pathway Is a Central Regulatory Node Linking Innate Immune Tolerance and Trained Immunity. Cell Metab. *29*, 211–220.e5.

Drakesmith, H., Nemeth, E., and Ganz, T. (2015). Ironing out ferroportin. Cell Metab. *22*, 777–787.

Drapier, J.C., and Hibbs, J.B., Jr. (1988). Differentiation of murine macrophages to express nonspecific cytotoxicity for tumor cells results in L-arginine-dependent inhibition of mitochondrial iron-sulfur enzymes in the macrophage effector cells. J. Immunol. *140*, 2829–2838.

Everts, B., Amiel, E., van der Windt, G.J., Freitas, T.C., Chott, R., Yarasheski, K.E., Pearce, E.L., and Pearce, E.J. (2012). Commitment to glycolysis sustains survival of NO-producing inflammatory dendritic cells. Blood *120*, 1422–1431.

Everts, B., Amiel, E., Huang, S.C., Smith, A.M., Chang, C.H., Lam, W.Y., Redmann, V., Freitas, T.C., Blagih, J., van der Windt, G.J., et al. (2014). TLR-driven early glycolytic reprogramming via the kinases TBK1-IKKε supports the anabolic demands of dendritic cell activation. Nat. Immunol. *15*, 323–332.

Gillen, K.M., Mubarak, M., Nguyen, T.D., and Pitt, D. (2018). Significance and *In Vivo* Detection of Iron-Laden Microglia in White Matter Multiple Sclerosis Lesions. Front. Immunol. *9*, 255.

Grootveld, M., Bell, J.D., Halliwell, B., Aruoma, O.I., Bomford, A., and Sadler, P.J. (1989). Non-transferrin-bound iron in plasma or serum from patients with idiopathic hemochromatosis. Characterization by high performance liquid chromatography and nuclear magnetic resonance spectroscopy. J. Biol. Chem. *264*, 4417–4422.

Gu, Z., Liu, T., Tang, J., Yang, Y., Song, H., Tuong, Z.K., Fu, J., and Yu, C. (2019). Mechanism of Iron Oxide-Induced Macrophage Activation: The Impact of Composition and the Underlying Signaling Pathway. J. Am. Chem. Soc. *141*, 6122–6126.

Haeggström, J.Z., and Funk, C.D. (2011). Lipoxygenase and leukotriene pathways: biochemistry, biology, and roles in disease. Chem. Rev. *111*, 5866–5898.

Hall, C.J., Boyle, R.H., Astin, J.W., Flores, M.V., Oehlers, S.H., Sanderson, L.E., Ellett, F., Lieschke, G.J., Crosier, K.E., and Crosier, P.S. (2013). Immunoresponsive gene 1 augments bactericidal activity of macrophage-lineage cells by regulating β-oxidation-dependent mitochondrial ROS production. Cell Metab. *18*, 265–278.

Hall, C.J., Sanderson, L.E., Lawrence, L.M., Pool, B., van der Kroef, M., Ashimbayeva, E., Britto, D., Harper, J.L., Lieschke, G.J., Astin, J.W., et al. (2018). Blocking fatty acid-fueled mROS production within macrophages alleviates acute gouty inflammation. J. Clin. Invest. *128*, 1752–1771.

Handa, P., Thomas, S., Morgan-Stevenson, V., Maliken, B.D., Gochanour, E., Boukhar, S., Yeh, M.M., and Kowdley, K.V. (2019). Iron alters macrophage polarization status and leads to steatohepatitis and fibrogenesis. J. Leukoc. Biol. *105*, 1015–1026.

Hider, R.C., and Hoffbrand, A.V. (2018). The Role of Deferiprone in Iron Chelation. N. Engl. J. Med. *379*, 2140–2150.

Hillar, M., Lott, V., and Lennox, B. (1975). Correlation of the effects of citric acid cycle metabolites on succinate oxidation by rat liver mitochondria and submitochondrial particles. J. Bioenerg. *7*, 1–16.

Holmes-Hampton, G.P., Ghosh, M.C., and Rouault, T.A. (2018). Methods for Studying Iron Regulatory Protein 1: An Important Protein in Human Iron Metabolism. Methods Enzymol. *599*, 139–155.

Infantino, V., Iacobazzi, V., Menga, A., Avantaggiati, M.L., and Palmieri, F. (2014). A key role of the mitochondrial citrate carrier (SLC25A1) in TNFα- and IFNγ-triggered inflammation. Biochim. Biophys. Acta *1839*, 1217–1225.

Kakhlon, O., Manning, H., Breuer, W., Melamed-Book, N., Lu, C., Cortopassi, G., Munnich, A., and Cabantchik, Z.I. (2008). Cell functions impaired by frataxin deficiency are restored by drug-mediated iron relocation. Blood *112*, 5219–5227.

Kang, H.M., Ahn, S.H., Choi, P., Ko, Y.A., Han, S.H., Chinga, F., Park, A.S., Tao, J., Sharma, K., Pullman, J., et al. (2015). Defective fatty acid oxidation in renal tubular epithelial cells has a key role in kidney fibrosis development. Nat. Med. *21*, 37–46.

Kelly, B., and O'Neill, L.A. (2015). Metabolic reprogramming in macrophages and dendritic cells in innate immunity. Cell Res. *25*, 771–784.

Kohler, S.A., Henderson, B.R., and Kühn, L.C. (1995). Succinate dehydrogenase b mRNA of Drosophila melanogaster has a functional iron-responsive element in its 5′-untranslated region. J. Biol. Chem. *270*, 30781–30786.

Krawczyk, C.M., Holowka, T., Sun, J., Blagih, J., Amiel, E., DeBerardinis, R.J., Cross, J.R., Jung, E., Thompson, C.B., Jones, R.G., and Pearce, E.J. (2010). Toll-like receptor-induced changes in glycolytic metabolism regulate dendritic cell activation. Blood *115*, 4742–4749.

Kroner, A., Greenhalgh, A.D., Zarruk, J.G., Passos Dos Santos, R., Gaestel, M., and David, S. (2014). TNF and increased intracellular iron alter macrophage polarization to a detrimental M1 phenotype in the injured spinal cord. Neuron *83*, 1098–1116.

Lampropoulou, V., Sergushichev, A., Bambouskova, M., Nair, S., Vincent, E.E., Loginicheva, E., Cervantes-Barragan, L., Ma, X., Huang, S.C., Griss, T., et al. (2016). Itaconate Links Inhibition of Succinate Dehydrogenase with Macrophage Metabolic Remodeling and Regulation of Inflammation. Cell Metab. *24*, 158–166.

Le, N.T., and Richardson, D.R. (2002). The role of iron in cell cycle progression and the proliferation of neoplastic cells. Biochim. Biophys. Acta *1603*, 31–46.

Li, C., Zhang, Y., Cheng, X., Yuan, H., Zhu, S., Liu, J., Wen, Q., Xie, Y., Liu, J., Kroemer, G., et al. (2018). PINK1 and PARK2 Suppress Pancreatic Tumorigenesis through Control of Mitochondrial Iron-Mediated Immunometabolism. Dev. Cell *46*, 441–455.e8.

Loenarz, C., and Schofield, C.J. (2008). Expanding chemical biology of 2-oxoglutarate oxygenases. Nat. Chem. Biol. *4*, 152–156.

Love, M.I., Huber, W., and Anders, S. (2014). Moderated estimation of fold change and dispersion for RNA-seq data with DESeq2. Genome Biol. *15*, 550.

Mackay, G.M., Zheng, L., van den Broek, N.J., and Gottlieb, E. (2015). Analysis of Cell Metabolism Using LC-MS and Isotope Tracers. Methods Enzymol. *561*, 171–196.

Mills, E.L., Kelly, B., Logan, A., Costa, A.S.H., Varma, M., Bryant, C.E., Tourlomousis, P., Dabritz, J.H.M., Gottlieb, E., Latorre, I., et al. (2016). Succinate Dehydrogenase Supports Metabolic Repurposing of Mitochondria to Drive Inflammatory Macrophages. Cell *167*, 457–470.e13.

Mills, E.L., Ryan, D.G., Prag, H.A., Dikovskaya, D., Menon, D., Zaslona, Z., Jedrychowski, M.P., Costa, A.S.H., Higgins, M., Hams, E., et al. (2018). Itaconate is an anti-inflammatory metabolite that activates Nrf2 via alkylation of KEAP1. Nature *556*, 113–117.

Murphy, M.P., and O'Neill, L.A.J. (2018). Krebs Cycle Reimagined: The Emerging Roles of Succinate and Itaconate as Signal Transducers. Cell *174*, 780–784.

Nairz, M., Schroll, A., Sonnweber, T., and Weiss, G. (2010). The struggle for iron - a metal at the host-pathogen interface. Cell. Microbiol. *12*, 1691–1702.

Nakazawa, M.S., Keith, B., and Simon, M.C. (2016). Oxygen availability and metabolic adaptations. Nat. Rev. Cancer *16*, 663–673.

Nathan, C., and Xie, Q.W. (1994). Nitric oxide synthases: roles, tolls, and controls. Cell *78*, 915–918.

Netz, D.J., Stith, C.M., Stümpfig, M., Köpf, G., Vogel, D., Genau, H.M., Stodola, J.L., Lill, R., Burgers, P.M., and Pierik, A.J. (2011). Eukaryotic DNA polymerases require an iron-sulfur cluster for the formation of active complexes. Nat. Chem. Biol. *8*, 125–132.

Núñez, G., Sakamoto, K., and Soares, M.P. (2018). Innate Nutritional Immunity. J. Immunol. *201*, 11–18.

Pagani, A., Nai, A., Corna, G., Bosurgi, L., Rovere-Querini, P., Camaschella, C., and Silvestri, L. (2011). Low hepcidin accounts for the proinflammatory status associated with iron deficiency. Blood *118*, 736–746.

Pandolfo, M., and Hausmann, L. (2013). Deferiprone for the treatment of Friedreich's ataxia. J. Neurochem. *126* (*Suppl 1*), 142–146.

Papathanassiu, A.E., Ko, J.H., Imprialou, M., Bagnati, M., Srivastava, P.K., Vu, H.A., Cucchi, D., McAdoo, S.P., Ananieva, E.A., Mauro, C., and Behmoaras, J. (2017). BCAT1 controls metabolic reprogramming in activated human macrophages and is associated with inflammatory diseases. Nat. Commun. *8*, 16040.

Papazyan, R., Sun, Z., Kim, Y.H., Titchenell, P.M., Hill, D.A., Lu, W., Damle, M., Wan, M., Zhang, Y., Briggs, E.R., et al. (2016). Physiological Suppression of Lipotoxic Liver Damage by Complementary Actions of HDAC3 and SCAP/SREBP. Cell Metab. *24*, 863–874.

Paul, B.T., Manz, D.H., Torti, F.M., and Torti, S.V. (2017). Mitochondria and iron: current questions. Expert Rev. Hematol. *10*, 65–79.

Peyssonnaux, C., Cejudo-Martin, P., Doedens, A., Zinkernagel, A.S., Johnson, R.S., and Nizet, V. (2007). Cutting edge: essential role of hypoxia inducible factor-1alpha in development of lipopolysaccharide-induced sepsis. J. Immunol. *178*, 7516–7519.

Recalcati, S., Locati, M., Marini, A., Santambrogio, P., Zaninotto, F., De Pizzol, M., Zammataro, L., Girelli, D., and Cairo, G. (2010). Differential regulation of iron homeostasis during human macrophage polarized activation. Eur. J. Immunol. *40*, 824–835.

Recalcati, S., Gammella, E., Buratti, P., Doni, A., Anselmo, A., Locati, M., and Cairo, G. (2019). Macrophage ferroportin is essential for stromal cell proliferation in wound healing. Haematologica *104*, 47–58.

Robinson, M.D., McCarthy, D.J., and Smyth, G.K. (2010). edgeR: a Bioconductor package for differential expression analysis of digital gene expression data. Bioinformatics *26*, 139–140.

Rudolf, J., Makrantoni, V., Ingledew, W.J., Stark, M.J., and White, M.F. (2006). The DNA repair helicases XPD and FancJ have essential iron-sulfur domains. Mol. Cell *23*, 801–808.

Selak, M.A., Armour, S.M., MacKenzie, E.D., Boulahbel, H., Watson, D.G., Mansfield, K.D., Pan, Y., Simon, M.C., Thompson, C.B., and Gottlieb, E. (2005). Succinate links TCA cycle dysfunction to oncogenesis by inhibiting HIF-alpha prolyl hydroxylase. Cancer Cell *7*, 77–85.

Sindrilaru, A., Peters, T., Wieschalka, S., Baican, C., Baican, A., Peter, H., Hainzl, A., Schatz, S., Qi, Y., Schlecht, A., et al. (2011). An unrestrained proinflammatory M1 macrophage population induced by iron impairs wound healing in humans and mice. J. Clin. Invest. *121*, 985–997.

Soares, M.P., and Hamza, I. (2016). Macrophages and Iron Metabolism. Immunity *44*, 492–504.

Sohn, Y.S., Breuer, W., Munnich, A., and Cabantchik, Z.I. (2008). Redistribution of accumulated cell iron: a modality of chelation with therapeutic implications. Blood *111*, 1690–1699.

Solinas, G., Borén, J., and Dulloo, A.G. (2015). De novo lipogenesis in metabolic homeostasis: more friend than foe? Mol. Metab. *4*, 367–377.

Subramanian, A., Tamayo, P., Mootha, V.K., Mukherjee, S., Ebert, B.L., Gillette, M.A., Paulovich, A., Pomeroy, S.L., Golub, T.R., Lander, E.S., and Mesirov, J.P. (2005). Gene set enrichment analysis: a knowledge-based approach for interpreting genome-wide expression profiles. Proc. Natl. Acad. Sci. USA *102*, 15545–15550.

Sumimoto, H. (2008). Structure, regulation and evolution of Nox-family NADPH oxidases that produce reactive oxygen species. FEBS J. *275*, 3249–3277.

Suragani, R.N., Zachariah, R.S., Velazquez, J.G., Liu, S., Sun, C.W., Townes, T.M., and Chen, J.J. (2012). Heme-regulated eIF2α kinase activated Atf4 signaling pathway in oxidative stress and erythropoiesis. Blood *119*, 5276–5284.

Szklarczyk, D., Franceschini, A., Wyder, S., Forslund, K., Heller, D., Huerta-Cepas, J., Simonovic, M., Roth, A., Santos, A., Tsafou, K.P., et al. (2015). STRING v10: protein-protein interaction networks, integrated over the tree of life. Nucleic Acids Res. *43*, D447–D452.

Tam, F.W., Smith, J., Morel, D., Karkar, A.M., Thompson, E.M., Cook, H.T., and Pusey, C.D. (1999). Development of scarring and renal failure in a rat model of crescentic glomerulonephritis. Nephrol. Dial. Transplant. *14*, 1658–1666.

Tannahill, G.M., Curtis, A.M., Adamik, J., Palsson-McDermott, E.M., McGettrick, A.F., Goel, G., Frezza, C., Bernard, N.J., Kelly, B., Foley, N.H., et al. (2013). Succinate is an inflammatory signal that induces IL-1β through HIF-1α. Nature *496*, 238–242.

Thompson, M.G., Corey, B.W., Si, Y., Craft, D.W., and Zurawski, D.V. (2012). Antibacterial activities of iron chelators against common nosocomial pathogens. Antimicrob. Agents Chemother. *56*, 5419–5421.

Tong, W.H., and Rouault, T.A. (2006). Functions of mitochondrial ISCU and cytosolic ISCU in mammalian iron-sulfur cluster biogenesis and iron homeostasis. Cell Metab. *3*, 199–210.

Tong, W.H., and Rouault, T.A. (2007). Metabolic regulation of citrate and iron by aconitases: role of iron-sulfur cluster biogenesis. Biometals *20*, 549–564.

Tong, W.H., Sourbier, C., Kovtunovych, G., Jeong, S.Y., Vira, M., Ghosh, M., Romero, V.V., Sougrat, R., Vaulont, S., Viollet, B., et al. (2011). The glycolytic shift in fumarate-hydratase-deficient kidney cancer lowers AMPK levels, increases anabolic propensities and lowers cellular iron levels. Cancer Cell *20*, 315–327.

Tong, W.H., Maio, N., Zhang, D.L., Palmieri, E.M., Ollivierre, H., Ghosh, M.C., McVicar, D.W., and Rouault, T.A. (2018). TLR-activated repression of Fe-S cluster biogenesis drives a metabolic shift and alters histone and tubulin acetylation. Blood Adv. *2*, 1146–1156.

Tyrakis, P.A., Yurkovich, M.E., Sciacovelli, M., Papachristou, E.K., Bridges, H.R., Gaude, E., Schreiner, A., D'Santos, C., Hirst, J., Hernandez-Fernaud, J., et al. (2017). Fumarate Hydratase Loss Causes Combined Respiratory Chain Defects. Cell Rep. *21*, 1036–1047.

Vinchi, F., Costa da Silva, M., Ingoglia, G., Petrillo, S., Brinkman, N., Zuercher, A., Cerwenka, A., Tolosano, E., and Muckenthaler, M.U. (2016). Hemopexin therapy reverts heme-induced proinflammatory phenotypic switching of macrophages in a mouse model of sickle cell disease. Blood *127*, 473–486.

Vinothkumar, K.R., Zhu, J., and Hirst, J. (2014). Architecture of mammalian respiratory complex I. Nature *515*, 80–84.

Weiss, J.M., Davies, L.C., Karwan, M., Ileva, L., Ozaki, M.K., Cheng, R.Y., Ridnour, L.A., Annunziata, C.M., Wink, D.A., and McVicar, D.W. (2018). Itaconic acid mediates crosstalk between macrophage metabolism and peritoneal tumors. J. Clin. Invest. *128*, 3794–3805.

Wickham, H. (2016). ggplot2: Elegant Graphics for Data Analysis (Springer-Verlag).

Williams, N.C., and O'Neill, L.A.J. (2018). A Role for the Krebs Cycle Intermediate Citrate in Metabolic Reprogramming in Innate Immunity and Inflammation. Front. Immunol. *9*, 141.

Yang, X., Xia, R., Yue, C., Zhai, W., Du, W., Yang, Q., Cao, H., Chen, X., Obando, D., Zhu, Y., et al. (2018). ATF4 Regulates $CD4^{+}$ T Cell Immune Responses through Metabolic Reprogramming. Cell Rep. *23*, 1754–1766.

Zanganeh, S., Hutter, G., Spitler, R., Lenkov, O., Mahmoudi, M., Shaw, A., Pajarinen, J.S., Nejadnik, H., Goodman, S., Moseley, M., et al. (2016). Iron oxide nanoparticles inhibit tumour growth by inducing pro-inflammatory macrophage polarization in tumour tissues. Nat. Nanotechnol. *11*, 986–994.

STAR★METHODS

KEY RESOURCES TABLE

REAGENT or RESOURCE	SOURCE	IDENTIFIER
Antibodies		
ATF-4 (D4B8) Rabbit mAb	Cell Signaling Technologies	Cat# 11815
Anti-SDHB	Abcam	Cat# ab14714
Anti- ACTB	Santa Cruz	Cat# sc-47778
Anti-rat ED1	Bio-Rad	Cat# MCA341R
anti-PCNA	Abcam	Cat# ab29
Polyclonal Rabbit Anti-mouse immunoglobulins/HRP	DAKO	Cat# P0260
Polyclonal Swine Anti-rabbit immunoglobulins/HRP	DAKO	Cat# P0217
Chemicals, Peptides, and Recombinant Proteins		
Recombinant Human M-CSF	PeproTech	Cat# AF-300-25
Oil-Red-O	Sigma	Cat# O0625
Haematoxylin	Sigma	Cat# MHS16
Iron(III) chloride ($FeCl_3$)	Sigma	Cat# 451649
Deferiprone (DEF)	Sigma	Cat# 379409
2-deoxyglucose (2-DG)	Sigma	Cat# D6134
NADP	Sigma	Cat# N5755
cis-aconitic acid	Sigma	Cat# A3412
MTT	Sigma	Cat# M5655
phenazine methosulfate	Sigma	Cat# P9625
isocitrate dehydrogenase	Sigma	Cat# I5036
Cerulenin	Sigma	Cat# C2389
Critical Commercial Assays		
Seahorse XF Cell Mito Stress Test Kit	Agilent Technologies	Cat #103015-100
RNeasy mini kit	QIAGEN	Cat# 74106
RNase-free DNase Kit	QIAGEN	Cat# 79254
NEBNext Ultra II Directional RNA Library Prep kit	NEB	Cat# E7760
iScript cDNA Synthesis Kit	Bio-Rad	Cat# 170-8891
Brilliant II SYBR Green QPCR Master Mix	Agilent	Cat# 600828
Pierce BCA Protein Assay Kit	Thermo Fisher Scientific	Cat# 23225
IL-1β ELISA	Invitrogen	Cat# 88-7261-88
TNFα ELISA	Invitrogen	Cat# 88-7346-88
Deposited Data		
RNA-sequencing data	This paper	GEO accession number GSE128885
Oligonucleotides		
Table S2	This paper	N/A
Software and Algorithms		
picard (v.2.6.0)	N/A	https://broadinstitute.github.io/picard/
edgeR (v.3.22.3)	(Robinson et al., 2010)	https://bioconductor.org/packages/release/bioc/html/edgeR.html
DESeq2 (v.1.14.1)	(Love et al., 2014)	https://bioconductor.org/packages/release/bioc/html/DESeq2.html
pcaExplorer (v.2.6.0)	N/A	https://github.com/federicomarini/pcaExplorer

(*Continued on next page*)

Continued

REAGENT or RESOURCE	SOURCE	IDENTIFIER
pheatmap (v 1.0.10)	N/A	https://cran.r-project.org/web/packages/pheatmap/index.html
ggplot2 (v.3.0.0)	(Wickham, 2016)	https://cran.r-project.org/web/packages/ggplot2/index.html
R/Bioconductor environment (v.3.4.4)	N/A	http://www.R-project.org/
Gene Set Enrichment Analysis (GSEA) (v5.2)	(Subramanian et al., 2005)	http://software.broadinstitute.org/gsea/index.jsp
STRING database	(Szklarczyk et al., 2015)	https://string-db.org/
GraphPad prism (v 7.02)	GraphPad Software	N/A
Other		
RPMI 1640 medium	Life Technologies	Cat# 11879020
Fetal calf serum (FCS)	Labtech International	Cat# FB-1001
Hanks' Balanced Salt Solution (HBSS)	Life Technologies	Cat# 14170112
Histopaque	Sigma	Cat# 1077
Seahorse XF RPMI medium	Agilent Technologies	Cat # 103576-100
SILAC medium	SILAC medium	Cat# A2494201
L-glutamine	Thermo Fisher Scientific	Cat# 25030081
L-Arginine	Sigma	Cat# A8094
L-Lysine	Sigma	Cat# L8662
D-Glucose U-13C, 99%	Cambridge isotope laboratories	Cat# CLM-1396
AMPure XP Beads	Beckman Coulter	Cat# A63880
SuperSignal West Femto Chemiluminescent Substrate	Thermo Fisher Scientific	Cat# 34580
TWEEN 20	Sigma	Cat# P1379
ON-TARGETplus SMARTpool - Human	Dharmacon	Cat# L-HUMAN-XX
DharmaFECT 1 Transfection Reagent	Dharmacon	Cat# T-2001
OPTIMEM	Thermo Fisher Scientific	Cat# 11058021

LEAD CONTACT AND MATERIALS AVAILABILITY

Further information and requests for resources and reagents should be directed to and will be fulfilled by the Lead Contact, Jacques Behmoaras (Jacques.behmoaras@imperial.ac.uk).

EXPERIMENTAL MODEL AND SUBJECT DETAILS

Rats

WKY (male, 16-weeks old, WKY/NCrl) rats used for NTN experiments were purchased from Charles River UK. All rats were used straight from the source by housing them until the appropriate experimental age. All procedures were performed in accordance to institutional guidelines and procedures approved by the UK Home Office (United Kingdom Animals Scientific Procedures Act, 1986).

CKD patients

Acute effects of intravenous iron administration were investigated in patients with stable non-immune chronic kidney disease, not requiring renal replacement therapy. Samples were provided after written informed consent, and in accordance with NHS Health Research authority approval. A summary of patient characteristics is provided in Table S1. All patients were clinically well, without evidence of acute infection or incurrent illness at the time of iron administration, and none had received intravenous iron or blood transfusion in the preceding three months.

METHOD DETAILS

Isolation of macrophages and PBMCs

Human monocyte-derived macrophages (hMDMs) were differentiated from buffy cones from healthy donors using gradient separation (Histopaque 1077, Sigma) and adhesion purification. Following Histopaque separation, peripheral blood mononuclear cells were

re-suspended in RPMI 1640 (Life Technologies), and monocytes were purified by adherence for 1 hour at 37°C, 5% CO_2. The monolayer was washed three times with HBSS to remove non-adherent cells, and monocytes were matured for 5 days in RPMI containing 100 ng/mL macrophage colony-stimulating factor (M-CSF, PeproTech, London, UK) and 10% fetal calf serum (Labtech International). For the deferiprone (DEF) treatment, macrophages were treated with 500 μM DEF (Sigma), in full culture media overnight. Cells were then prepared for either RNA-sequencing or LC-MS experiments using D-Glucose U-^{13}C. For rescue experiments involving the addition of $FeCl_3$, macrophages deprived from iron with over-night DEF treatment, were treated with $FeCl_3$ (200 μM, Sigma) for 8 hours, after which the cells were isolated and used in different assays. Lipopolysaccharide (LPS, Sigma) treatment of hMDMs was used either for RNA-seq or LC-MS experiments.

CKD patients received intravenous iron, as Ferinject (ferric carboxymaltose, 50mg/mL) at a total dose of 1-1.5g by slow infusion over 30 minutes. Peripheral blood was drawn in EDTA immediately prior and 120 min following iron infusion, and processed immediately. Peripheral blood mononuclear cells (PBMCs) were isolated by Histopaque (1077, Sigma) density gradient centrifugation and used for ATF4 Western Blotting before and after iron infusion.

Metabolic extracellular flux analysis

Real-time measurements of OCR and ECAR were performed using a Seahorse XF96 Extracellular Flux Analyzer (Agilent Technologies). hMDMs cultured for 5 days in presence of RPMI containing M-CSF (100 ng/ml) and FCS (10%) were washed and incubated with DEF. Cells were then washed, re-suspended using the non-enzymatic cell dissociation buffer (Sigma) and 5×10^5 hMDMs were seeded as a monolayer in a 96-well microplate containing Seahorse XF RPMI medium (Agilent Technologies). The different metabolic drugs were injected (oligomycin 1μM, FCCP 2μM, rotenone/antimycin 1μM, 2-DG 50mM) during real-time measurements of OCR and ECAR, using the Seahorse XF Cell Mito Stress Test Kit (Agilent Technologies). Basal respiration was calculated as the last measurement before addition of oligomycin – non mitochondrial respiration (minimum rate measurement after Rot/AntA). Maximal respiration is shown as the maximum rate measurement after addition of FCCP – non mitochondrial respiration. Estimated ATP production designates the last measurement before addition of oligomycin – minimum rate after oligomycin. Glycolysis refers to ECAR values before the addition of oligomycin.

Stable isotope tracing by liquid chromatography-mass spectrometry (LC-MS)

For stable isotope tracing, human macrophages (treated or left as basal) were incubated for 8 hours with SILAC medium lacking glucose and phenol red (GIBCO) with addition of L-glutamine (0.5 mM), arginine (200 mg/L), lysine (40 mg/L) and uniformly labeled glucose (2 g/L D-Glucose U-^{13}C, 99%, Cambridge isotope laboratories). Cells were then washed three times with PBS and 200μl of extraction buffer (50% LC-MS grade methanol and 30% acetonitrile, 20% ultrapure water) was added per 10^6 cells. Following 15 min incubation in dry ice, the cells were scraped off and kept under vigorous shaking for 15 min at 4°C, and left for 1 hour incubation at −20°C. Following centrifugation, the supernatant was stored at −80°C until further analysis. For the cell supernatant analysis, the same procedure was performed in supernatant obtained from 10^6 cells. For the steady-state metabolomics analysis, cells were stimulated with LPS and DEF or left unstimulated (basal) at indicated times, followed by the same extraction procedure described above.

Samples were randomized in order to avoid bias due to machine drift and processed blindly. LC-MS analysis was performed using a Q Exactive mass spectrometer (Thermo Fisher Scientific) coupled to a Dionex U3000 UHPLC system. The liquid chromatography system was fitted with a Sequant ZIC-pHILIC column (150 mm × 2.1 mm) and guard column (20 mm × 2.1 mm) from Merck Millipore (Germany) and temperature maintained at 40°C. The mobile phase was composed of 20 mM ammonium carbonate and 0.1% ammonium hydroxide in water (solvent A), and acetonitrile (solvent B). The flow rate was set at 200 μL/min with the gradient as described previously (Mackay et al., 2015). The mass spectrometer was operated in full MS and polarity switching mode. The acquired spectra were analyzed using XCalibur Qual Browser and XCalibur Quan Browser software (Thermo Fisher Scientific). To generate the heatmap, the raw data were normalized by total ion count and missing values were replaced by half of the minimum value of each metabolite. Heatmap was then generated with the heatmap.2 function of the gplots package by creating z-score values and using default clustering methods.

RNA extraction and library preparation

Total RNA was extracted from hMDMs using Trizol (Invitrogen) and RNeasy mini kit (QIAGEN) according to manufacturer's instructions, with an additional purification step by on-column DNase treatment using the RNase-free DNase Kit (QIAGEN) to ensure elimination of any genomic DNA. The integrity and quantity of total RNA was determined using a NanoDrop 1000 spectrophotometer (Thermo Fisher Scientific) and Agilent 2100 Bioanalyzer (Agilent Technologies). 500 ng of total RNA was used to generate RNA-seq libraries using NEBNext Ultra II Directional RNA Library Prep kit from Illumina, according to the manufacturer's instructions. Briefly, RNA was purified and fragmented with poly-T oligo-attached magnetic beads, using two rounds of purification followed by the first and second cDNA strand synthesis. Next, cDNA 3′ ends were adenylated and adapters ligated followed by 11 cycles of library amplification. The libraries were size selected using AMPure XP Beads (Beckman Coulter), subsequently purified and their quality was checked using Agilent 2100 Bioanalyzer. Samples were randomized to avoid batch effects and multiplexed libraries were run on a single lane (8 samples/lane) of the HiSeq 2500 platform (Illumina) to generate 100bp paired-end reads.

Bioinformatics

An average depth of 52 M reads per sample was achieved. Sequencing adapters were removed using Trimmomatic (v.0.36) and the reads quality was checked using FastQC (v.0.11.2) before and after trimming. Reads were aligned to the human genome (GRCh38.primary_assembly.genome.fa; annotation: gencode.v25.annotation.gtf) using tophat2 package (v.2.1.0: -b2-sensitive,–library-type fr-firststrand). An average mapping percentage of 97.1% was achieved and the average number of properly paired reads was 51.4 M (~95%). Mapping quality, read distribution, gene body coverage, GC content and rRNA contamination, were checked using picard (v.2.6.0) software. Gene level read counts were computed using HT-Seq-count (v.0.6.1) with strict "-m intersection-strict" mode. Genes with less than 10 aligned reads across all samples were filtered out as lowly expressed genes, resulting in 15,732 expressed genes. Differential gene expression analysis between groups was performed using DESeq2 (v.1.14.1) and significantly differentially expressed genes were reported using a cut-of fold-change (FC) at 1.5 and below 1% Benjamini-Hochberg (BH) adjusted P-value (P_{adj}). Unsupervised hierarchical clustering and principal component analysis (PCA) were performed using pcaExplorer (v.2.6.0, https://github.com/federicomarini/pcaExplorer) and pheatmap (v 1.0.10, https://cran.r-project.org/web/packages/pheatmap/index.html) packages respectively. Volcano plots of differentially expressed genes were generated using ggplot2 (v.3.0.0, https://cran.r-project.org/web/packages/ggplot2/index.html) package. All raw RNA-seq data processing steps were performed in Cx1 high-performance cluster computing environment, Imperial College London. Further analyses were conducted in R/Bioconductor environment v.3.4.4 (http://www.R-project.org/).

Gene Set Enrichment Analysis (GSEA v5.2, http://software.broadinstitute.org/gsea/index.jsp) was utilized to identify potential specific biological pathway or signature enrichment between different treatment groups. All gene sets with an FDR less than 0.25 were considered as statistically significant. For gene ontology (GO) analysis, a cut-of (FC > 1.5, $P_{adj} < 0.01$) was applied to the differentially expressed genes and the resulting transcripts were entered to STRING database (https://string-db.org/) to interrogate protein-protein interactions together with pathway enrichement.

Quantitative RT-PCR

Total RNA was extracted from human macrophages using the TRIzol reagent (Invitrogen) according to the manufacturer's instructions, and cDNA was synthesized using iScript cDNA Synthesis Kit (Bio-Rad). A total of 10 ng cDNA for each sample was used. All quantitative RT-PCRs were performed on a ViaA 7 Real-Time PCR System (Life Technologies) using Brilliant II SYBR Green QPCR Master Mix (Agilent), followed by ViiA 7 RUO Software for the determination of Ct values. Results were analyzed using the comparative Ct method, and each sample was normalized to the reference mRNA level of *HPRT* gene, to account for any potential cDNA loading differences.

Western blotting

Human macrophages were lysed in Laemmli sample buffer supplemented with protease inhibitors and resolved by SDS-PAGE, transferred into PVDF membranes, and subjected to immunoblotting with the primary antibodies against ATF4 (Cell Signaling Technologies), SDHB (Abcam), ACTB (Santa Cruz) and secondary detection antibodies. The probed proteins were detected using SuperSignal West Femto Chemiluminescent Substrate (Thermo Fisher Scientific Inc., Rockford, IL).

RNA interference

hMDMs were re-plated in six-well plates (1×10^6 cells per well) in RPMI (Invitrogen) overnight and transfected with siGENOME SMARTpool for human *ISCU* (100 nM, Dharmacon SMART pool) or non-targeting siRNA pool as the scrambled control siRNA using Dharmafect 1 (1:50, Dharmacon) as a transfection reagent in OPTIMEM medium (Invitrogen). Following 8 h incubation with OPTIMEM media containing either *ISCU* or non-targeting siRNA, cells were washed and further cultured for 48 h in presence of RPMI media containing M-CSF and FCS. Iron supplementation was achieved by culturing the macrophages for an additional 8 hours in presence of $FeCl_3$.

ELISA

Detection of human IL-1β and TNFα (Invitrogen) in hMDM culture supernatants at indicated conditions, were performed by sandwich ELISA, using technical duplicates, following the manufacturer's recommendations. Light absorbance was measured using Multiscan Ascent (Thernofisher).

Nephrotoxic Nephritis and immunohistochemistry

Male WKY rats (Charles River UK) weighing 300-360 g were used for the induction of nephrotoxic nephritis (NTN). Nephrotoxic serum was prepared in rabbits and NTN was induced by intravenous injection as previously described (Tam et al., 1999). Groups of animals were given either DEF (200 mg/kg/day in 0.1% carboxymethyl cellulose by oral gavage) or vehicle only. Rats were individually housed in metabolic cages overnight for urine collection, and urinary protein levels were determined by sulphosalicylic acid method. Serum blood urea nitrogen and creatinine were measured using an Abbott Architect c8000 clinical chemistry analyzer. Paraffin-embedded kidneys were sectioned for periodic acid-Schiff (PAS) staining. Fibrin deposition was scored as the number of glomerular quadrants involved from '0' (absence of fibrin) to '4+' (fibrin occupying more than $^3/_4$ of the glomerulus) in 50 consecutive glomeruli. Crescent formation was recorded as presence/absence in 100 consecutive glomeruli and presented as percentage of glomeruli with

crescents. Immunohistochemistry was performed and developed with EnVision+ System-HRP (Dako) using mouse anti-rat ED1 (Bio-Rad) and anti-PCNA [PC10] (Abcam) antibodies. Pictures of 20 consecutive glomeruli from each ED1 stained slide were taken with a QImaging Retiga 2000R Scientific CCD Camera, using Image-Pro® Plus Version 7.0 software. The size of the glomeruli and ED1 stained area in glomeruli were measured using ImageJ software. The number of anti-PCNA stained cells in 20 consecutive glomeruli were counted. All histological and immunohistochemical analyses were performed in a blinded manner.

Oil-red-o staining

Optimal Cutting Temperature compound embedded frozen rat kidneys were sectioned at 5 microns thickness and 10% formalin fixed for 10 min at room temperature prior to staining. hMDMs cultured in slide-chambers were treated with DEF alone (500 μM) or together with $FeCl_3$ (200 μM) or cerulenin (20 μM) for 48 hours. The cells were fixed with 10% formalin at 4°C. Kidney sections and hMDMs were stained for lipids by Oil-Red-O (0.5% Oil red O dye in 60% isopropanol; Sigma) and counterstained with Haematoxylin (Sigma). Pictures were obtained by light microscopy using a Leica DM6 B upright microscope and a Leica DFC7000 T camera.

Aconitase in-gel assay

In-gel aconitase activities were measured as described previously (Holmes-Hampton et al., 2018; Tong and Rouault, 2006). Aconitase activity gels are composed of a separating gel containing 8% acrylamide (132 mM Tris base, 132 mM borate, 3.6mM citrate) and a 4% acrylamide (67 mM Tris base, 67 mM borate, 3.6 mM citrate) stacking gel. The running buffer contains 25 mM Tris pH 8.3, 192 mM glycine, and 3.6 mM citrate. Protein concentration of macrophage lysates was measured by using the Pierce BCA Protein Assay Kit and the sample buffer contained 25 mM Tris-Cl, pH 8.0, 10% glycerol, and 0.025% bromophenol blue. Electrophoresis was carried out at 180 V, 4°C, for 2 hours. Aconitase activities were assayed by incubating the gel in the dark at 37°C in 100 mM Tris (pH 8.0), 1 mM NADP, 2.5 mM cis-aconitic acid, 5 mM $MgCl_2$, 1.2 mM MTT, 0.3 mM phenazine methosulfate, and 5 U/ml isocitrate dehydrogenase (Sigma).

QUANTIFICATION AND STATISTICAL ANALYSIS

Data are presented as mean ± s.e.m. and analyzed using GraphPad Prism software (version 7.02; GraphPad). One-way ANOVA (followed by Dunnett' or Tukey's multiple comparison tests) and Student's t test were performed unless otherwise stated. Differences in percentage of fold change following *ISCU* siRNA knockdown were tested for significance using a one-sample-t test. In uniformly labeled [U]-13C-glucose experiments, one-way ANOVA was applied to test for significance in glucose-derived isotopologues.

DATA AND CODE AVAILABILITY

The accession number for the RNA-sequencing data reported in this paper is GEO accession number GEO: GSE128885.

Cell Reports, Volume *28*

Supplemental Information

Acute Iron Deprivation Reprograms Human Macrophage Metabolism and Reduces Inflammation *In Vivo*

Marie Pereira, Tai-Di Chen, Norzawani Buang, Antoni Olona, Jeong-Hun Ko, Maria Prendecki, Ana S.H. Costa, Efterpi Nikitopoulou, Laura Tronci, Charles D. Pusey, H. Terence Cook, Stephen P. McAdoo, Christian Frezza, and Jacques Behmoaras

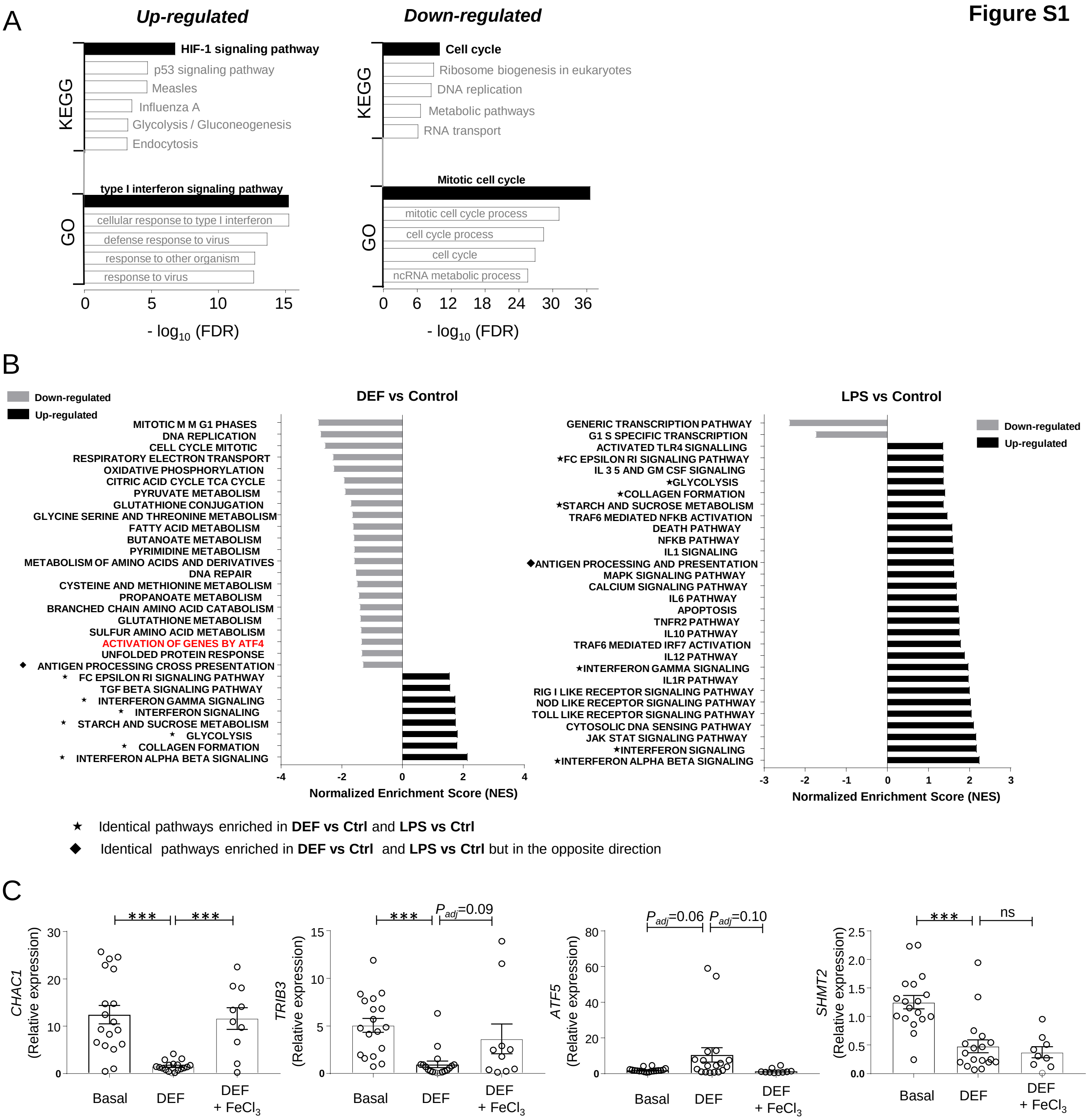

Figure S1. **Acute iron deprivation causes a distinct transcriptome and mediates an ATF4-dependent transcriptional response in human macrophages**. Related to Figure 1. (A) KEGG and Gene ontology (GO) analyses on differentially expressed genes (DEF vs Ctrl) in human macrophages. (B) Gene Set Enrichment Analysis (GSEA), on DEF (500μM, 24h) vs Ctrl and LPS (3h, 100ng/ml) vs Ctrl comparisons in human macrophages. ‘Activation of genes by ATF4’ pathway is shown in red. (C) ATF4 target gene expression in iron-deprived (DEF) and rescued (DEF+$FeCl_3$) human macrophages. ***, P<0.001 by ANOVA followed by Tukey’s multiple comparisons test. P_{adj} denotes adjusted *P*-value. FDR, false discovery rate. Error bars represent SEM.

Figure S2

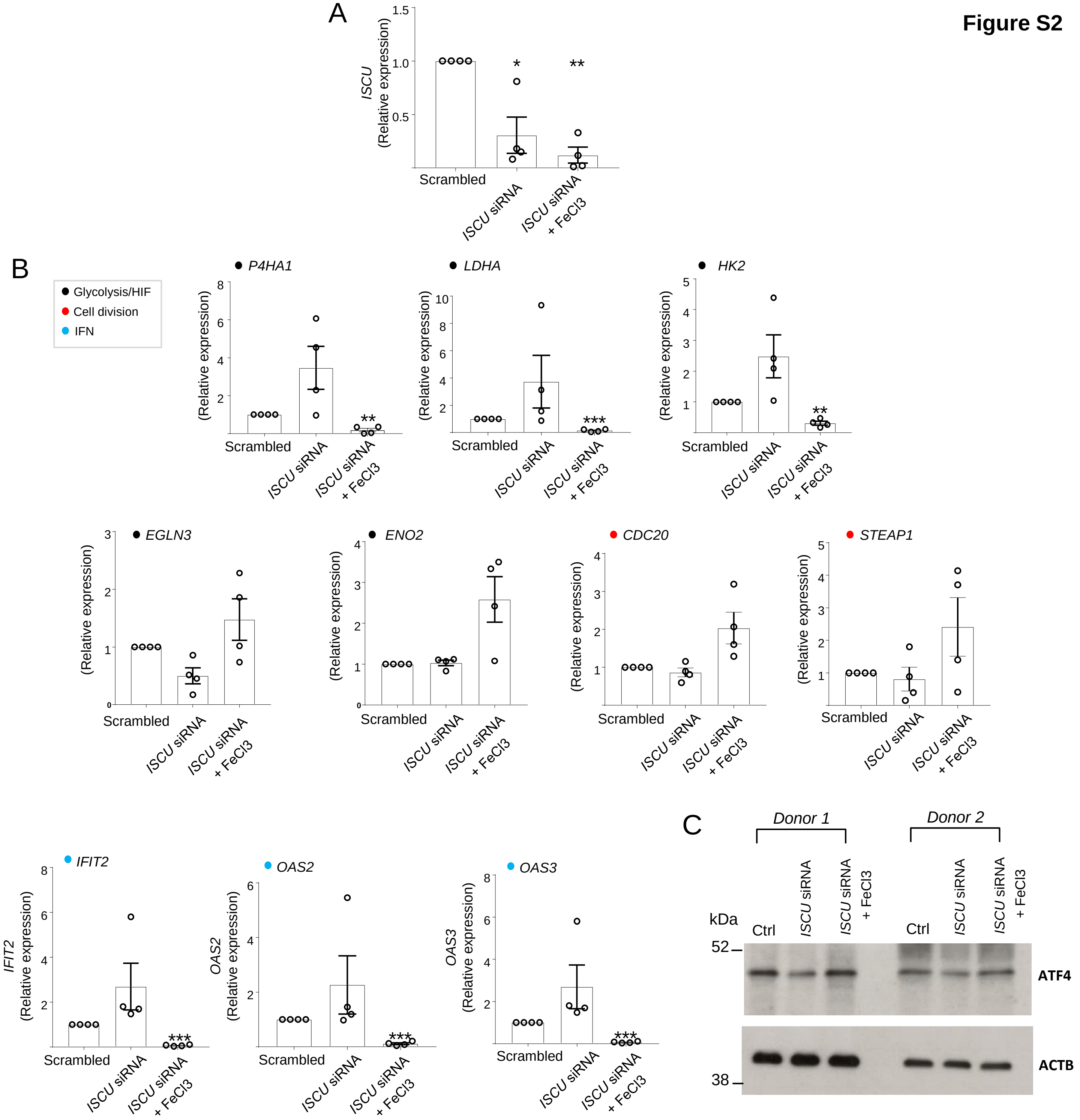

Figure S2. ***ISCU* mRNA knockdown causes similar transcriptional responses to the ones following deferiprone stimulation in human macrophages**. Related to Figure 1C and 1D. (A) *ISCU* expression by qRT-PCR. Human macrophages from 4 donors (n=4) were either treated with *ISCU* or scrambled siRNA. Iron replenishment was obtained by adding 200 µM $FeCl_3$ for 8 hours (*ISCU* siRNA + $FeCl_3$) following *ISCU*-siRNA. (B) qRT-PCR for glycolysis/HIF, cell cycle/mitosis and IFN genes (colour-coded). (C) ATF4 Western Blotting in *ISCU* siRNA and ISCU siRNA followed by iron replenishment (*ISCU* siRNA + $FeCl_3$) in 2 donors. *, P<0.05; **, P<0.01, ***, P<0.001 by one sample t-test compared to 100% (Scrambled). Error bars represent SEM.

Figure S3

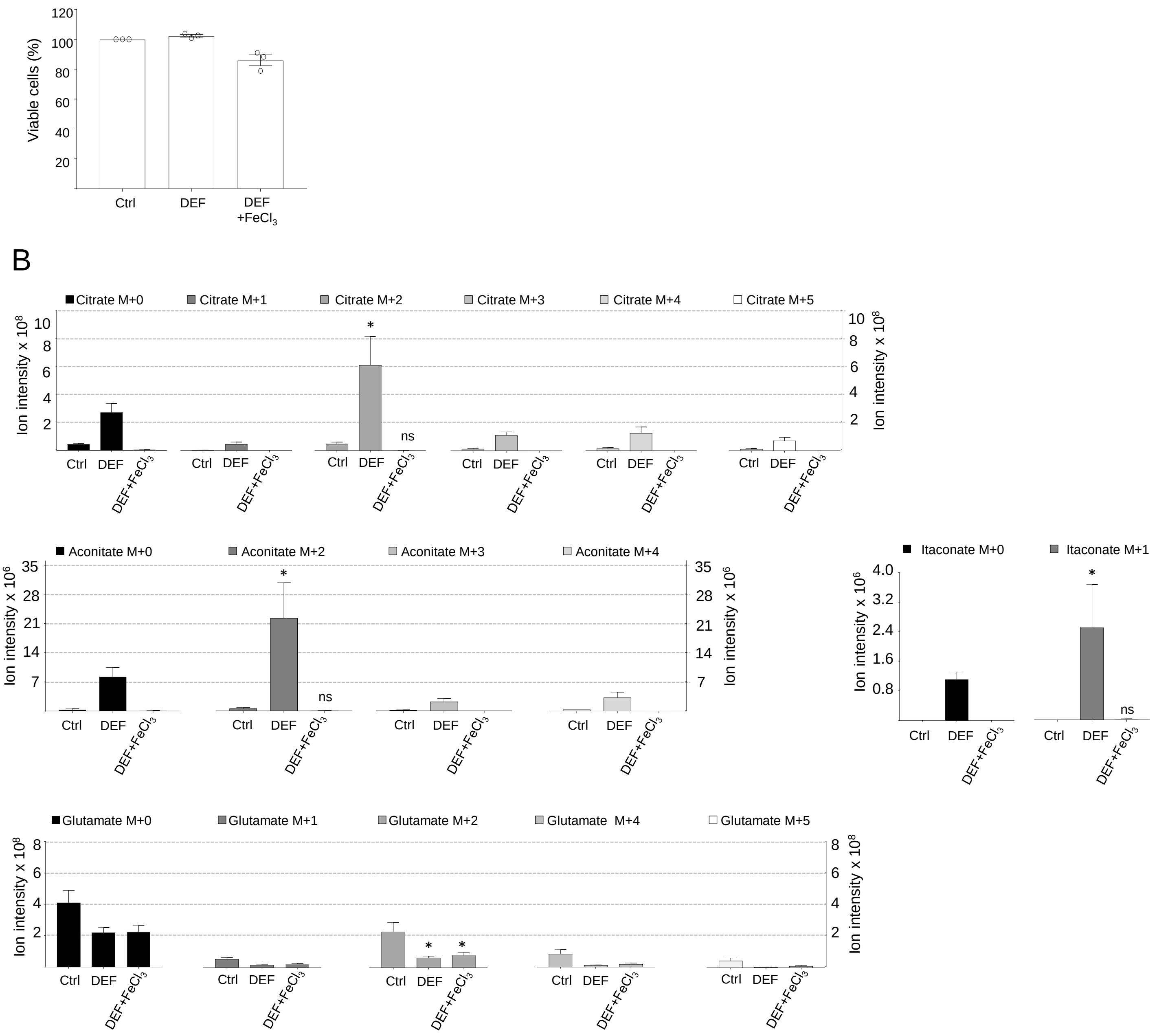

Figure S3. **Acute iron deprivation causes citrate, aconitate and itaconate accumulation while decreasing glutamate levels in human macrophages**. Related to Figure 3. (A) Cell viability measured by Alamar blue. n=3 donors. (B) Isotopologue quantification by LC-MS for citrate, aconitate, itaconate and glutamate. n=6 donors. *, $P<0.05$, when compared to control by ANOVA followed by Dunnett's multiple comparisons test. For simplicity, only glucose-derived isotopologues were compared for significance (M+2 citrate, glutamate, aconitate and M+1 itaconate). Error bars represent SEM.

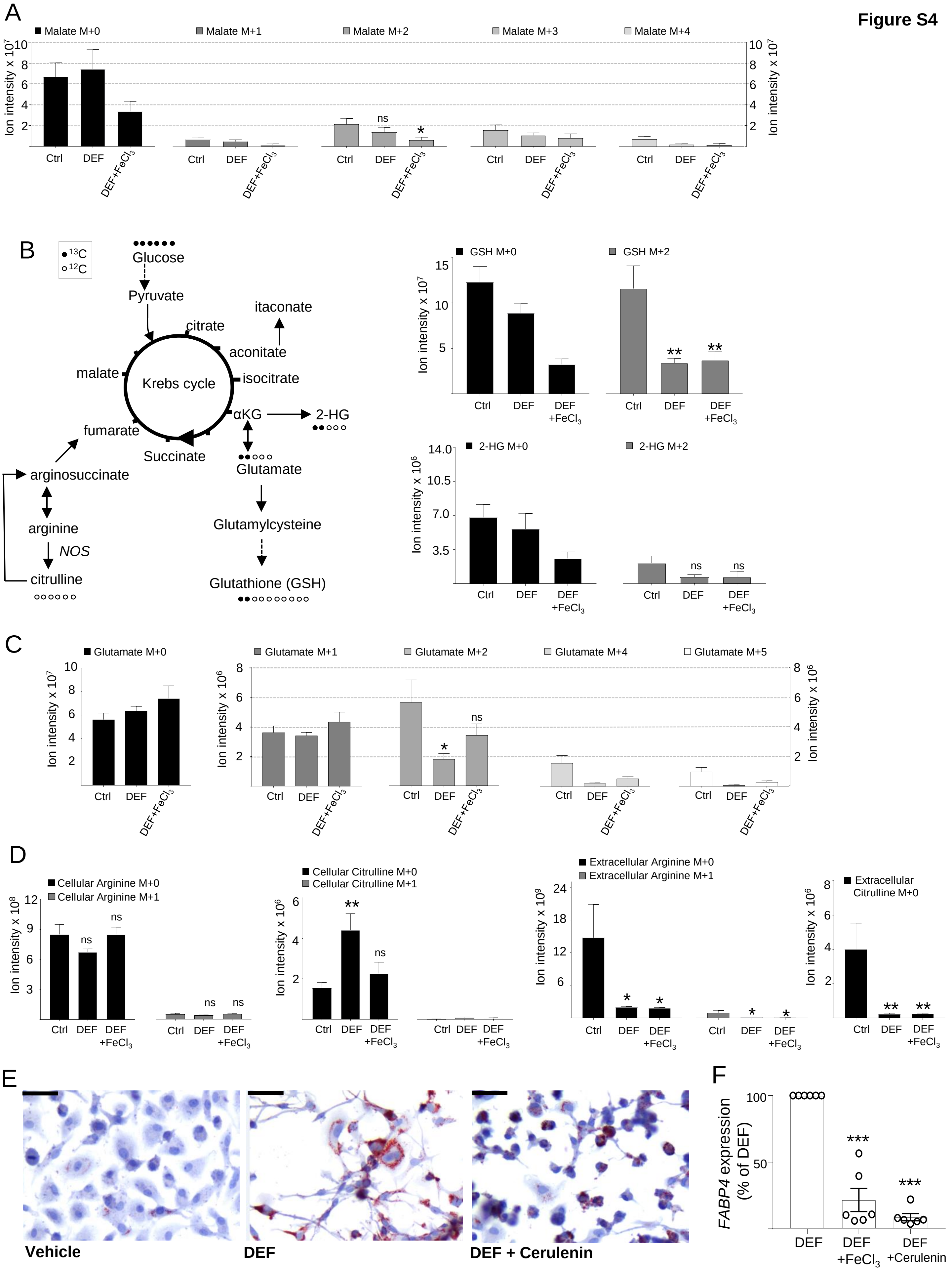
Figure S4
A
Malate M+0
Malate M+1
Malate M+2
Malate M+3
Malate M+4
Ion intensity x 10^7
Ctrl
DEF
DEF+FeCl3
ns
*
B
13C
12C
Glucose
Pyruvate
citrate
itaconate
aconitate
malate
Krebs cycle
isocitrate
αKG
2-HG
fumarate
Succinate
Glutamate
arginosuccinate
arginine
NOS
Glutamylcysteine
citrulline
Glutathione (GSH)
GSH M+0
GSH M+2
2-HG M+0
2-HG M+2
**
C
Glutamate M+0
Glutamate M+1
Glutamate M+2
Glutamate M+4
Glutamate M+5
Ion intensity x 10^6
D
Cellular Arginine M+0
Cellular Arginine M+1
Ion intensity x 10^8
Cellular Citrulline M+0
Cellular Citrulline M+1
Extracellular Arginine M+0
Extracellular Arginine M+1
Ion intensity x 10^9
Extracellular Citrulline M+0
E
Vehicle
DEF
DEF + Cerulenin
F
FABP4 expression (% of DEF)
***
DEF
DEF +FeCl3
DEF +Cerulenin

Figure S4. **Acute iron deprivation causes a brake in the TCA cycle, alters the urea cycle and induces *de novo* lipid accumulation**. Related to Figure 3. (A) Quantification of malate isotopologues by LC-MS in control (Ctrl), iron-deprived (DEF, 500µM), iron-rescued human macrophages with 200µM $FeCl_3$ supplementation (DEF+$FeCl_3$, 8h); n=6 donors. (B) Schematic illustration of glucose-αKG-derived metabolites and urea cycle in uniformly labelled [U]-^{13}C- glucose catabolism (left panel) and glutathione (GSH), 2-hydroxyglutarate (2-HG) M+0 and M+2 isotopologues (right panel) in control (Ctrl), iron-deprived (DEF), iron-rescued human macrophages with $FeCl_3$ supplementation (DEF+$FeCl_3$, 8h); n=6 donors. (C) Extracellular glutamate isotopologues quantified by LC-MS; n=6 donors. (D) Cellular (upper panel) and extracellular (lower panel) citrulline and arginine isotopologues quantified by LC-MS; n=6 donors. (E) Vehicle, DEF (500µM) or concomitant DEF and cerulenin (20µM)-treated human macrophages stained for oil-red-o. (F) *FABP4* expression (% of DEF) in hMDMs (n=2 donors). In (A), (B) and (C), only glucose-derived M+2 istopologues were compared for significance (M+2 GSH, M+2 2-HG, M+2 glutamate). *, P<0.05; **, P<0.01 compared to Control (Ctrl) by ANOVA followed by Dunnett’s multiple comparisons test. ***, P<0.001 by one sample t-test compared to 100% (DEF). ns, non-significant. Scale bars, 40µm. Error bars represent SEM.

Figure S5

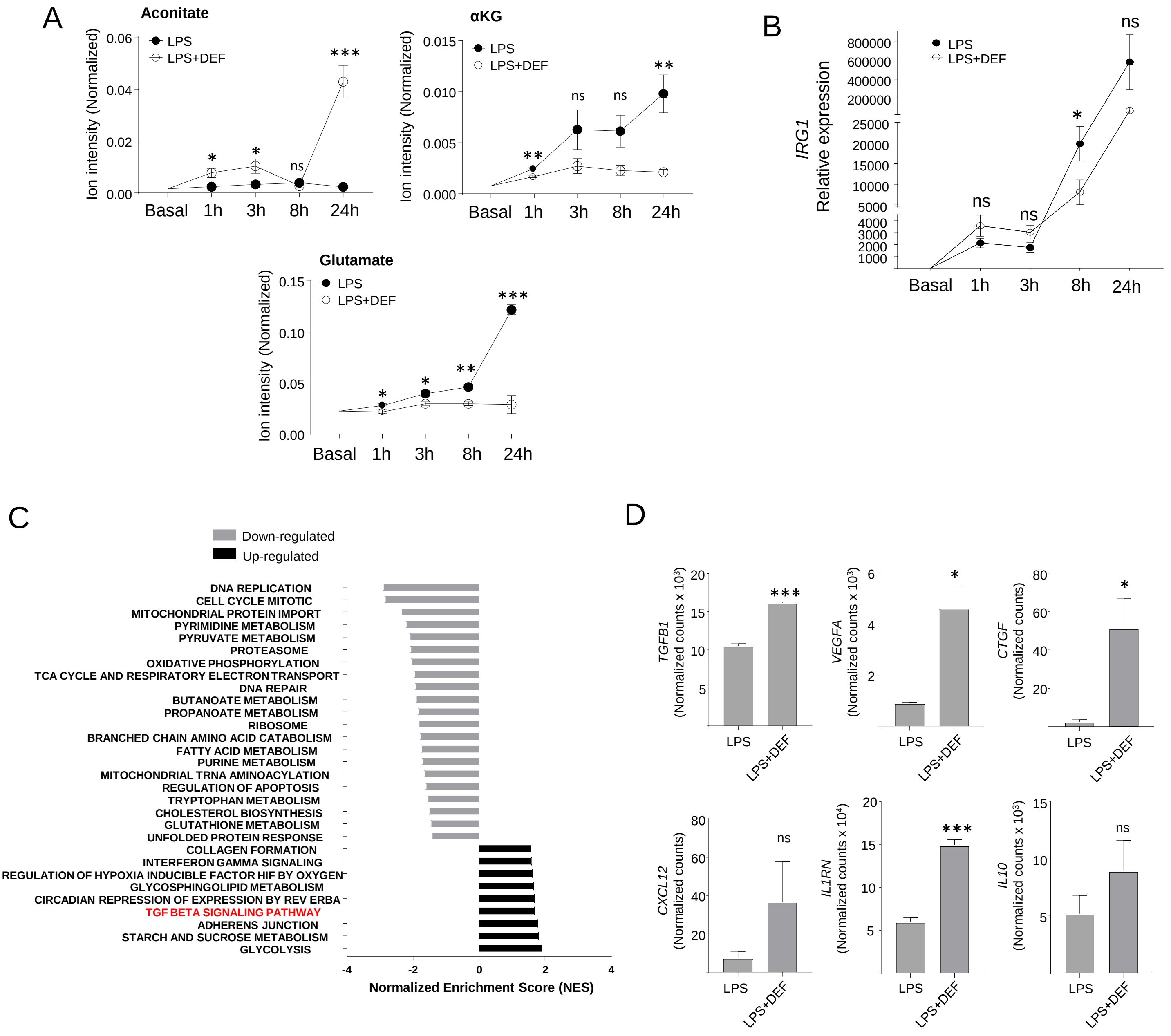

Figure S5. **Acute iron chelation limits pro-inflammatory macrophage polarization and promotes TGF-β signalling pathway**. Related to Figure 4. (A) LC-MS for aconitate, α-ketoglutarate (αKG) and glutamate in basal and LPS-treated (100ng/ml) human macrophages throughout a time course. LPS+DEF refers to macrophages pre-treated with DEF (500µM, 24h) prior to the LPS time-course; n=4 donors/group. (B) *IRG1* mRNA measured by qRT-PCR in basal and DEF-treated cells (500µM, 24h) throughout an LPS (100ng/ml) time course; n=4 donors/group. (C) Gene Set Enrichment Analysis for LPS+DEF vs. LPS comparison. (LPS, 3h, 100ng/ml; DEF, 24h, 500µM). TGFβ signalling pathway is shown in red. (D) *TGFB1*, *VEGFA*, *CTGF*, *CXCL12*, *IL1RN*, *IL10* expression measured by RNA-seq (normalized counts) in LPS and LPS+DEF stimulated human macrophages; n=3 donors/group. *, P<0.05; **, P<0.01; ***, P<0.001 by t-test; ns, non-significant. Error bars represent SEM.

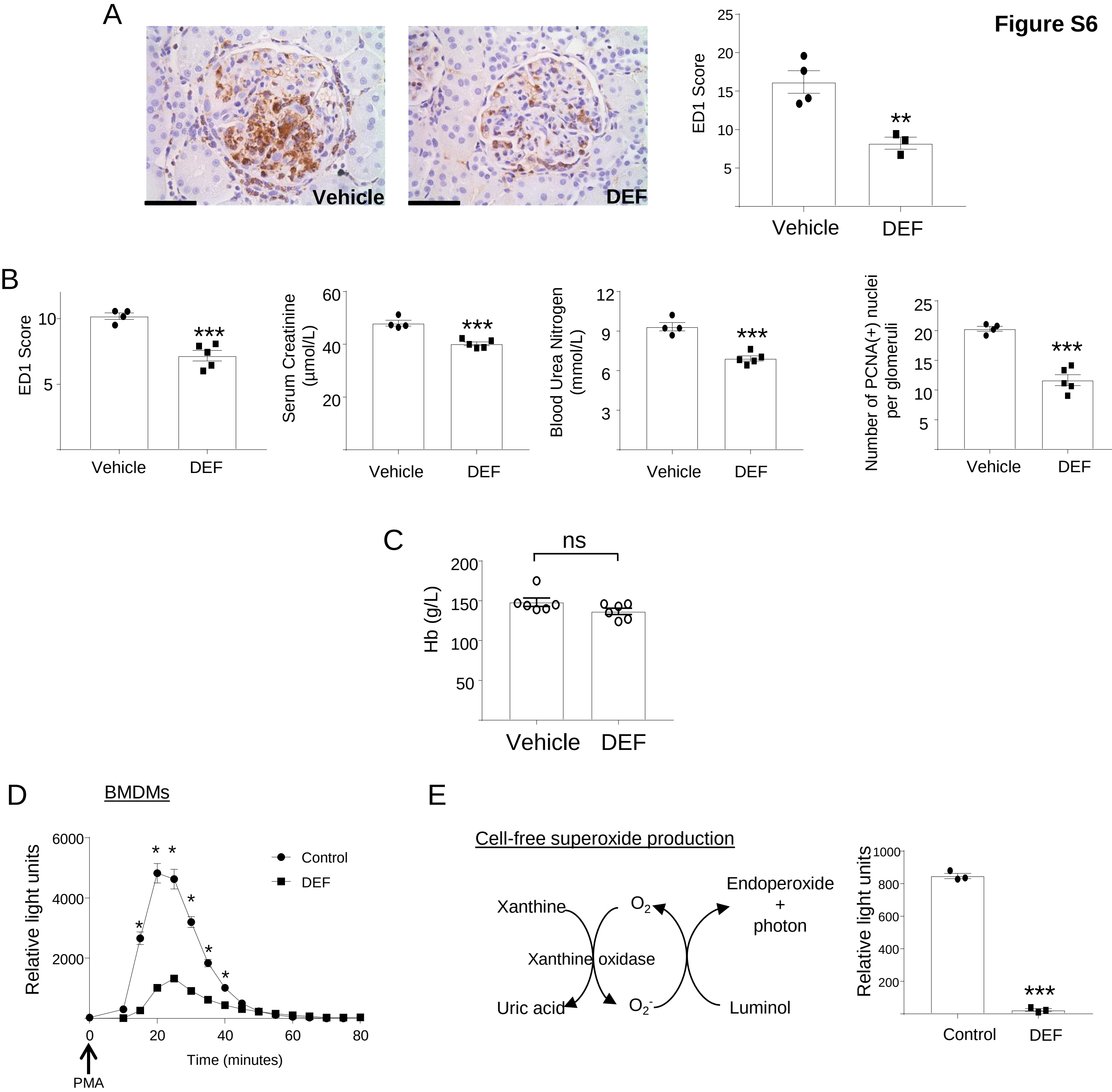

Figure S6. **Acute iron chelation decreases the severity of glomerulonephritis and reduces superoxide production in macrophages**. Related to Figure 6. (A) ED1 staining showing macrophages in the glomeruli; ED1 score (right panel) was normalized to glomerular size; at least n=3 rats/group; preventive NTN experiment. (B) ED1 score, serum creatinine, blood urea nitrogen, glomerular PCNA+ nuclei; at least n=4 rats/group; therapeutic NTN experiment. (C) Serum haemoglobin (Hb) counts; n=6 rats/group. (D) Superoxide chemiluminescence signal in WKY BMDMs stimulated with 1 µM phorbol myristate acetate (PMA) with or without of deferiprone (DEF, 500 µM); at least 3 rats/group. (E) Superoxide chemiluminescence signal produced by xanthine-xanthine oxidase reaction in a cell free setup (left panel) with or without deferiprone (DEF, 500 µM, right panel). *, P<0.05; **, P<0.01; ***, P<0.001 by t-test. ns, non-significant. Scale bars, 40µ. Error bars represent SEM.

	Patient 1	*Patient 2*	*Patient 3*	**Normal Ranges**
**Age**	77	83	51	
**Gender**	F	M	M	
**Cause of CKD**	Diabetes	Diabetes	Diabetes	
**Creatinine (µmol/L)**	272	302	456	60-125
**eGFR (ml/min/1.73m^2)**	16.2	18.5	13.9	>90
**CKD Stage**	IV	IV	V	
**Hb (g/dL)**	10.2	9.6	10.3	13-16.5
**Ferritin (µg/L)**	281	30	106	20-300
**Iron (µmol/L)**	9	N/A	11	12-31
**Transferrin Saturation (%)**	20	N/A	24	16-55
**Transferrin (g/L)**	1.8	N/A	1.8	1.7-3.4
**MCV (fL)**	98.7	87.2	89	83.5-99.5
**CRP (mg/L)**	3	19.1	0.9	0-5
**ESA**	Aranesp	No	No	

Table S1. **Clinical characteristics of chronic kidney disease (CKD) patients undergoing intravenous iron administration.** Related to Figure 1. eGFR, estimated glomerular filtration rate; Hb, haemoglobin; MCV, mean corpuscular volume; CRP, c-reactive protein; ESA, erythropoiesis stimulating agent.

Gene name / siRNA name	*Sequence*
***PLOD2*-F**	GGGAATGGACTTTTGCCGTC
***PLOD2*-R**	CCACAGCTTTCCATGACGAG
P4HA1-F	TTTGCACGGAAAGATGAGCC
***P4HA1*-R**	TCCTTCTCCACTGGCAAACA
***LDHA*-F**	GTGGAGGTTGTGCATGTTGT
***LDHA*-R**	CGTCAGAGGTGGCAGAACTA
***HK2*-F**	GCCATCCTGCAACACTTAGG
***HK2*-R**	CCACACCCACTGTCACTTTG
***STEAP1*-F**	TGCCTGGATTGAGCATGATG
***STEAP1*-R**	AATGCGTGTATTGTGCCCAG
***CDC20*-F**	ACCATGATGTTCGGGTAGCA
***CDC20*-R**	AATGTCTGCAGAGGAACCCA
***DDX21*-F**	AACTTCAAGTGGGCAAGCTG
***DDX21*-R**	CAGGGGCTACATCAGGTTCT
***EGLN3*-F**	CCTCACTGAAGACTGACCGT
***EGLN3*-R**	ACCACACACAAGACAGGGAT
***ENO2*-F**	CCCGACACCTGTATTGCATG
***ENO2*-R**	TGGGAGCCAAGAAGAGGATG
***SDHB*-F**	CACAGCTCCCCGTATCAAGA
***SDHB*-R**	CAAGAGCCACAGATGCCTTC
***NDUFS6*-F**	TCGGTTTGTAGGTCGTCAGA
***NDUFS6*-R**	CTAGTGGTGGTGCTGTCTGA
**CHAC1-F**	GACTTCATGCAGCTCTGTGG
**CHAC1-R**	TCTGTCTTGTGCACTGGAGT
***TRIB3*-F**	AGCTCACTCTGGGAACTGTG
***TRIB3*-R**	ATCCTGTCCCTCAACCTTGG
***ATF5*-F**	GGACCGCAAGCAAAAGAAGA
***ATF5*-R**	CTCGATGAGCAGGTCCTTGA
***SHMT2*-F**	CTGACTGCTCGACTTTTCCG
***SHMT2*-R**	GTGAGTAGTGGTGGTGACGA
***ISCU*-F**	ATCCTAGAAACGTGGGGTCC
***ISCU*-R**	CTTCCTCCACCGTCTTTCCT
***IFIT2*-F**	GCGAAACAACTGCTCCATCT
***IFIT2*-R**	CCAAGACATGCAAAGCCTCA
***OAS2*-F**	GCTTCCAACTCATCCACGTC
***OAS2*-R**	GGCAGGCTCAGAAGGAAAAG
***OAS3*-F**	TGCCATATTGACAGCCTCCA
***OAS3*-R**	GGATATGTGTGTGGCAGCAG
***IRG1*-F**	TGTGTGGACTTCATGAGCCT
***IRG1*-R**	TTAAACCCACCCATTCCCCA
***FABP4*-F**	AAACTGGTGGTGGAATGCGT
***FABP4*-R**	GCGAACTTCAGTCCAGGTCA
**SMARTpool siRNA *ISCU***	GAGCUAUGAGAUACGCACA<br>CAGCAUGUGGUGACGUAAU<br>CAUAAAACAGAUUGCGCAU<br>GGUCUGAAUAUUUGAUAGA

Table S2. **Primer sequences used in the study**. Related to STAR Methods. Gene names are followed by -F (Forward) and -R (Reverse). Individual siRNA target sequences are shown for the SMARTpool.
